# Supplementary material for: Clusia genomes shed light on the evolution and diversity of crassulacean acid metabolism physiotypes
Source: Nat Commun. 2026 May 5;17:3937. doi: 10.1038/s41467-026-71958-z (PMC13144421; doi:10.1038/s41467-026-71958-z)
Supplement: Supplementary file 1 — Supplementary Information [file 41467_2026_71958_MOESM1_ESM.pdf]

Clusia genomes shed light on the evolution and diversity of crassulacean acid metabolism physiotypes

Kramml and Herpell *et al.*

Supplementary Discussion 1. Taxonomic ambiguities and physiological phenotyping of *Clusia* spp.

Photosynthetic physiotypes of C_3+CAM such as weak CAM, inducible/facultative CAM, and CAM-cycling have long fueled a debate on the evolutionary constraints of CAM, as have the prospects for engineering CAM into C_3 plants. Understanding the biochemical and genetic factors governing physiotype diversity of CAM might lead to novel opportunities for engineering this pathway into non-CAM plants[1]. One of the major questions is how the physiological reprogramming necessary for CAM has evolved, but also what targets to focus on. Here, we sequenced the first genomes within clusioids representing the major physiotypes obligate and facultative CAM, as well as a C_3-like mode of photosynthesis. Based on genome information, physiological and multiomics analysis we provide evidence that polyploidization followed by diploidization might have played a major role in the evolution of CAM physiotypes in different *Clusia* species. We determined that C. *major* performs C_3+CAM and is not an exclusive and obligate CAM plant as previously described, due to taxonomic misidentification of the studied collections (see below). In contrast to previous studies, we demonstrated that WGD enabled sub-, neo-, and non-functionalization of duplicated gene pairs. Genic diploidization leads to shifts in plastidic CAM and starch-related pathways, which would explain the physiological diversity associated with the starch/sugar-malate cycle and the establishment of subtypes of CAM. A transition from a strong CAM to a weak CAM physiotype might represent the evolutionary trajectory from CAM to the energetically favorable C_3 physiotype but this needs further investigations. It is likely that the existence of plants representing intermediate stages of these evolutionary processes can lead to novel niche colonization and stabilization. This could explain the outstanding physiotypic diversity of plants within the genus *Clusia*[2] as well as their individual physiological plasticity and may be a good starting point in the discovery of breeding targets to generate plants with higher WUE. Whole-genome duplication coupled with fractionation and transposon-mediated genomic changes have shaped the ongoing process of diploidization and the emergence of extant photosynthetic modes in *Clusia* spp., and possibly beyond.

Here, we provide some additional discussion points regarding the taxonomic identification and associated uncertainties in *Clusia* that would exceed the scope of the main text but are quite

worthy of being closely considered. We also discuss the physiological phenotyping that we conducted to support our claims and, last but not least, we provide Supplementary Figures and Tables referenced in the main text.

Taxonomy of the *Clusia* spp. selected for this study

The diversity of physiotypes observed in the genus *Clusia* Plum. ex L. presents a possibility to understand the mechanisms of the evolution of CAM photosynthesis, therefore we selected species with contrasting physiological phenotypes for the study. The taxonomy of *Clusia* is notoriously challenging, partly because most species are vegetatively similar and the flowers are dioecious and variable, exhibiting variation in flower merosity even within the same sex. Differences between species are often subtle, or morphologically similar species have evolved independently in distantly related groups. Furthermore, due to their CAM photosynthesis, specimens are difficult to dry and preserve in herbarium collections, which is why they have not received adequate sampling and taxonomic attention in the past. As a result, most regional floras remained incompletely known[3,4], identification keys are missing for most groups or regions, and misidentifications are common in herbaria, living collections and the literature. Currently, however, knowledge of the genus *Clusia* is improving rapidly, taxonomy and species concepts are being refined[5,6] and new species are constantly being described[7–15] eventually cumulating in new floral or monographic treatments.

A decade ago, our laboratory received a backup of the large *Clusia* culture collection originally curated by Ulrich Lüttge, Technical University of Darmstadt. Over the years, we have utilized this collection extensively, during which we compiled a substantial dataset of physiological traits for a large variety of species. For the present study, we selected three different species exhibiting different types of photosynthetic modes: *C. rosea* Jacq. (obligate strong CAM), *C. minor* L. (reversible CAM induction), and *C. major* L. (consistent C_3-like behavior). The identification of these plants was confirmed or revised by consulting floras and other relevant literature[4,16,17] and by comparison with authentic specimens in the collection of the herbarium of the Natural History Museum Vienna (e.g., W0356736, W0391745, W0391747).

C. rosea (voucher W0356382) is easily characterized, even vegetatively, among others, by its sulphury yellow latex and thick, coriaceous obovate leaves lacking resin-ducts[4,17]. *C. rosea* is widespread and occurs from Florida and the Antilles south to Venezuela and Ecuador. It is generally accepted that *C. rosea* constitutively performs strong CAM[18–20].

C. minor (voucher W0391623) is a more challenging identification, since the group is among the least known and new species are still described or resurrected from synonymy[9]. *C. minor* in the broad or "traditional" sense is easily characterized by its exfoliating bark, smaller, narrower leaves, few-flowered inflorescences, usually dioecious flowers in which the pistillate flowers bear a resinous staminodial ring [4,16,17]. The plants studied here belong to the apomictic form with light tan (vs. reddish in *C. minor* s.str.) bark, ovoid (vs. globose) fruits and the staminodial ring lacking anthers (vs. a few present), to which the name *C. pratensis* Seem. applies[16]. *C. minor* s.str. is distributed in the West Indies and Central America, whereas *C. pratensis* is found in one isolated population in Costa Rica, and from Panama south to northern South America. Unfortunately, no conclusive consensus has been reached in the form of floral treatments or a comprehensive revision of the taxonomy of the entire *C. minor* group. In addition, the lectotype of *C. minor*, a pre-Linnean illustration from the Codex Boerhaavianus[21], actually shows a plant with ovoid fruits and contradicts the current use of the name for the taxon with globose fruits[9,16]. Therefore, the names *C. pratensis* and *C. minor* (in the sense of the lectotype) probably refer to the same species, and it remains to be seen whether the species with globose fruits, currently known as *C. minor* (s.str.), has ever been formally described. Additionally, the ITS sequence data used to distinguish between species of the *C. minor* group[6,9,14,22] is inconclusive and does not resolve relationships, likely due to a high level of hybridization between species in this clade (**Fig. 1a**). Therefore, a broad concept of *C. minor* (i.e., *C. minor* s.l.) is followed here until the group becomes better known. Nevertheless, all studied taxa in the *C. minor* complex show the capability of facultative CAM and also exhibit weak CAM under well-watered conditions[20,23–25].

C. major (voucher W0391620), was initially cultivated in our collection under the name *C. multiflora* Kunth, but we revised this identification after molecular analyses first revealed inconsistencies between nuclear markers of the specimen when compared to reference sequences of *C. multiflora*[26] (**Fig. 1a**). The plant carried molecular markers identical to those published as *C. venosa* Jacq., which is a synonym of *C. minor*[27]. Yet, it did not cluster with our own sequences of *C. minor* nor with sequences in public databases, suggesting a misidentification in literature, further supported by clear morphological distinctions from *C. minor*. The numerous, continuous resin channels of the leaves also exclude *C. multiflora* which has intermittent resin channels. When the specimen flowered for the first time, the plant could

be re-identified as *C. major*. *C. major* is characterized, among others, by subsessile leaves with prominent wavy resin lines, few-flowered inflorescences, and drooping creme-colored pistillate flowers with a ring of few free slender staminodes secreting resin at the tips[17,22]. Flower biology has not been studied, but the flowers in cultivation are apparently nocturnal, a rare feature in the genus. *C. major* is endemic to the Lesser Antilles and Trinidad.

Taxonomic misidentification of photosynthetic physiotypes

It is generally recognized that the subgenus *Clusia* to which both *C. major* and *C. rosea* belong has ancestrally developed CAM[28] (**Fig. 1a**). Our specimen of *C. major* consistently performed C_3-type photosynthesis with an additional weak CAM component. The revision of species identifications in related literature revealed surprising support for this observation.

Using molecular markers and morphological traits (e.g. leaf morphology), we traced and revisited the identification of *C. major* through available references. One accession identified as *C. venosa* (voucher W0356384) in the Lüttge collection was reported to show C_3-type physiology with the ability to perform weak CAM[29], and is here re-identified as *C. major* based on leaf morphology and molecular data (**Fig. 1a**). Furthermore, the closest relative of *C. major*, *C. aripoensis* Britton has been reported to also induce weak CAM[30]. Historically, *C. major* and *C. rosea* were often considered conspecific, with both names at times treated as the accepted name[3,21] contributing to considerable confusion. Given the prevailing taxonomic uncertainty in the genus, we also analyzed a further accession frequently reported in literature as *C. major* (voucher W0356387), which was reported to perform strong CAM[29]. Its reference sequence fell within the *C. rosea* clade (**Fig. 1a**) and sulphury yellow latex and leaf morphology support that identification therefore revising the physiotype reported for *C. major*.

Both above-mentioned cultivated accessions have been cited in several publications over the past 35 years including extensive data for physiological characterization[29,31] and evolutionary assumptions about CAM[32,33]. In the light of misidentifications, this data aligns with our own results from gas-exchange and titratable acidity measurements under comparable experimental conditions, particularly with respect to key CAM-inducing factors such as day/night temperature variation and water vapor pressure deficit[34] (**Supplementary Table 3**).

Physiological phenotyping

In controlled eco-chambers we measured titratable acidity (TA), net CO_2 fixation and stomatal conductance over a 24-hour period under simulated natural growth conditions where water availability was strictly monitored for precise daily irrigation of individual plants (**Supplementary Fig. 1ab**). *C. major* performs a C_3-like photosynthesis mode in combination with a weak CAM component under drought conditions, denoted by the slight nocturnal CO2 uptake (**Fig. 1b**). *C. rosea* exhibits a strong CAM physiotype, however, the substantial amount of CO_2 uptake at dawn (phase II), dusk (phase IV), and to some extent throughout the day (phase III) suggests that this species is capable of performing a fair amount of C_3 photosynthesis under certain conditions.

Complementary measurements of titratable acidity (TA) under well-watered and drought conditions further reflect these high oscillations at dawn/dusk (phase II/IV) in the three species investigated (**Supplementary Fig. 1c**). While the variability among highly controlled biological replicates differ to some extent, *C. rosea* consistently exhibits large nocturnal acidification of leaves under both conditions, indicating near-constitutive CAM. In contrast, *C. major* and *C. minor* show non-significant to moderate fluctuations at night under water-limited conditions. Large nighttime acid accumulation and significant daytime deacidification in both *C. major* and *C. minor*, however, consistently indicate an induction of CAM under drought conditions. Rather than discrete categories, our species or even individuals occupy different positions along a CAM continuum, from weak/facultative to strong constitutive expression, in any case encompassing the ability to perform CAM (**Supplementary Fig. 1d, Fig. 1c**).

The apparent uncertainties in both taxonomy and physiotype classification have likely confounded otherwise valid results in the existing literature. To address this issue and promote clarity in future work, we provide the first reference genomes of *Clusia* here (as opposed to single marker sequences), with digital voucher specimens for all revised and sequenced taxa (**Supplementary Table 1**), establishing a new reference framework amid persistent taxonomic uncertainty: *C. major* (W0391620, W0356417), *C. minor* s.l. (W0391623, W0356410), and *C. rosea* (W0356382).

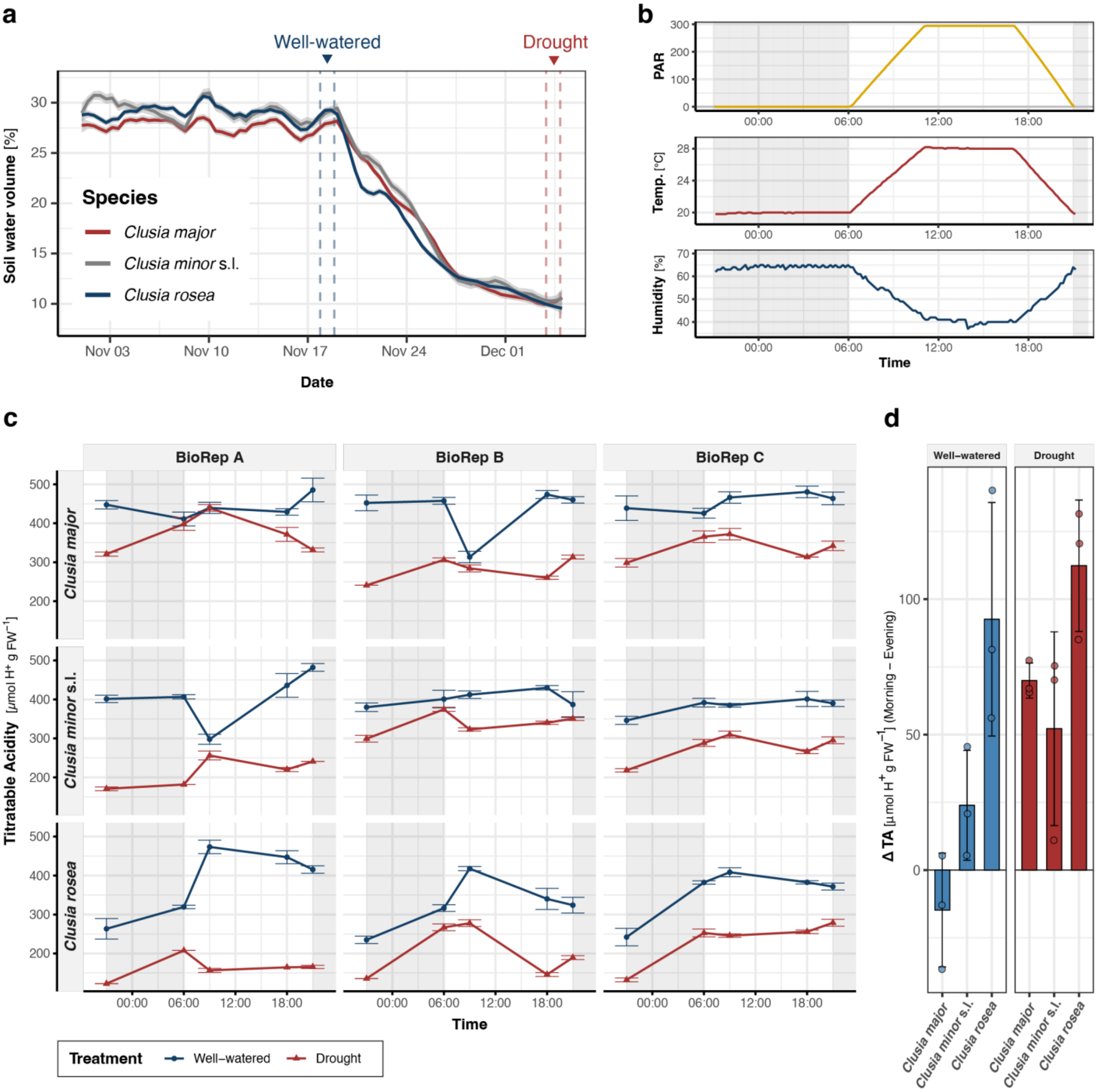

Supplementary Figure 1 | Physiological phenotyping of *Clusia major*, *C. minor* s.l., and *C. rosea*. a Watering scheme of nine biological replicates incubated for 6 weeks at 25-30% soil water volume (excerpt). Sampling under well-watered conditions was performed at five timepoints over a full 24-hour cycle (dashed lines). Irrigation of the same individuals was then gradually reduced to slowly reach a soil water volume of exactly 10%. After two weeks, a second sampling was performed under water-limited conditions (drought). **b** Highly controlled environmental / microclimatic parameters following a diurnal cycle as shown for light intensity (top), ambient temperature (middle), and relative humidity (bottom). **c** Titratable acidity measurements over a full 24-hour cycle of three individuals of *C. major* (top), *C. minor* s.l. (middle), and *C. rosea* (bottom). Each sample was measured three times using photometric assays (n=3). Error bars represent the ±standard deviation. **d** Nocturnal fluctuation (6am - 9pm) in titratable acidity (TA) averaged over technical triplicates for three biological replicates per species and treatment (n=3). Error bars represent the ±standard deviation. Source data are provided as a Source Data file. Rather than discrete categories, the species or even individuals occupy different positions along a CAM continuum, from weak/facultative to strong constitutive expression, in any case encompassing the ability to perform CAM.

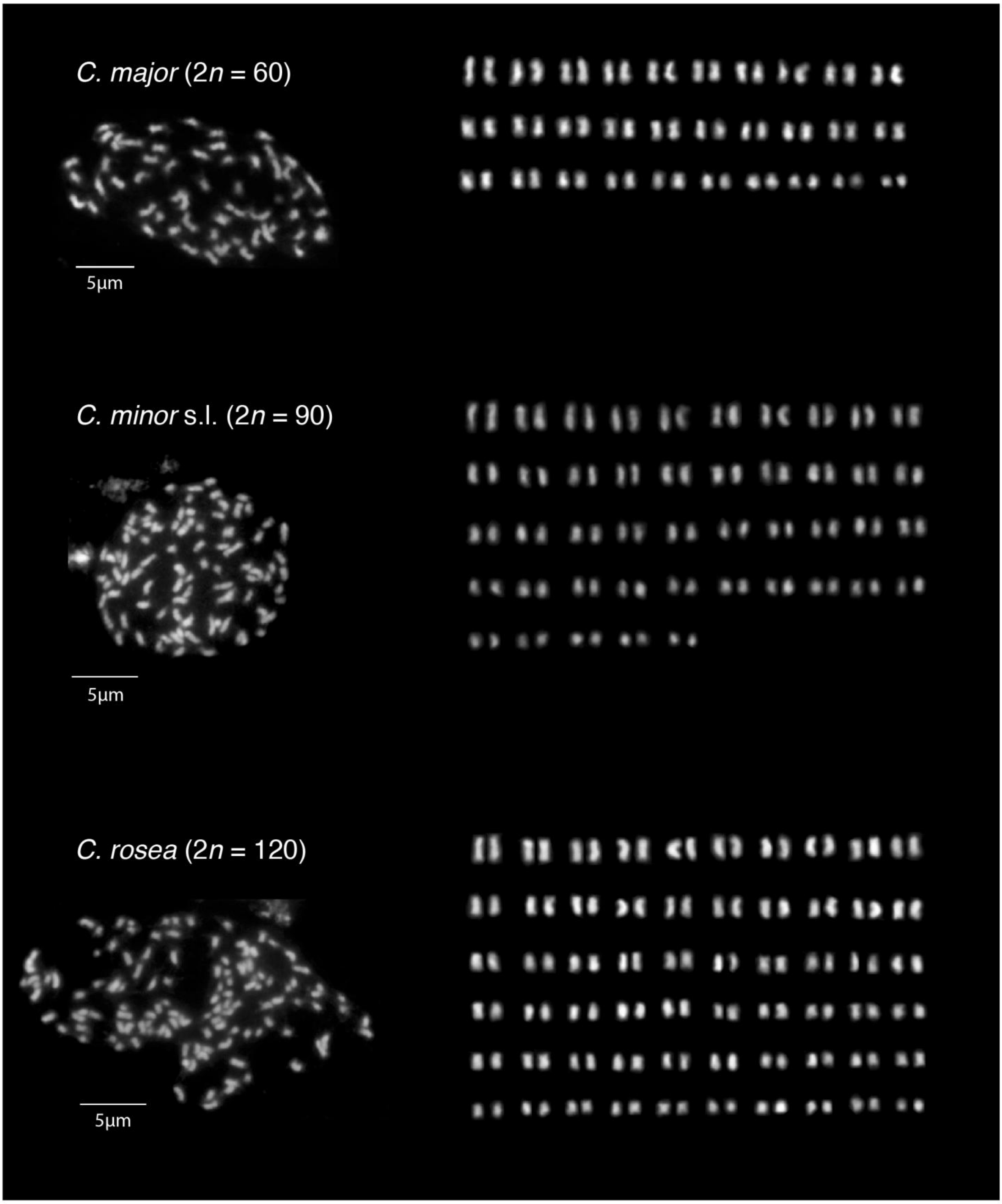

Supplementary Figure 2 | Chromosome numbers and karyotypes of *Clusia major* (2*n* = 60), *C. minor* s.l. (2*n* = 90) and *C. rosea* (2*n* = 120). Chromosome numbers unambiguously indicate that the three genomes differ in their ploidy levels.

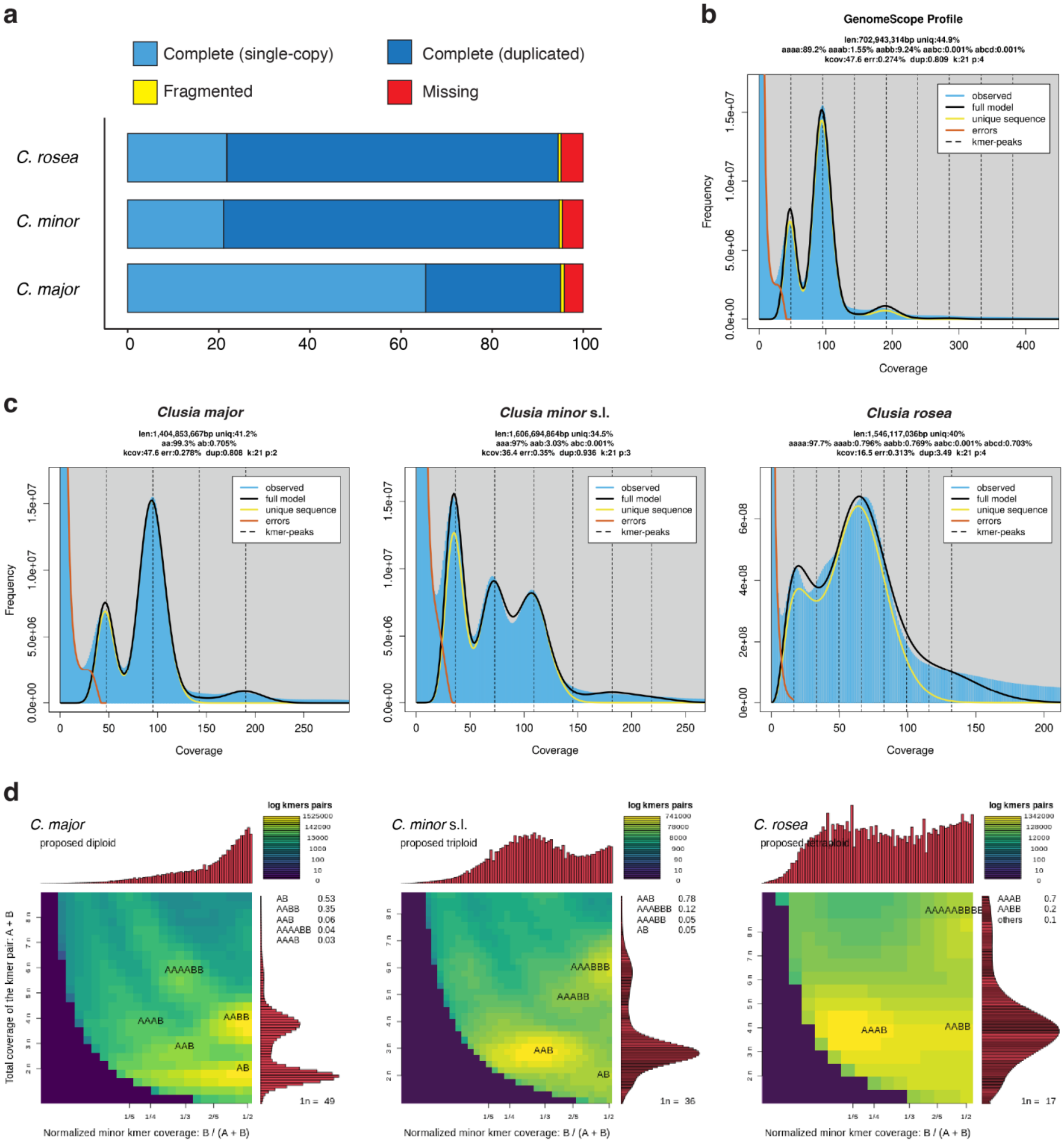

Supplementary Figure 3 | Assembly completeness and read-based ploidy level estimations of *Clusia* spp. a BUSCO scores of draft genomes on contig level (v1) using the lineage dataset of Eudicots (odb10). **b** Genomic properties based on GenomeScope for a tetraploid model of *Clusia major*. The high 2x peak in the k-mer spectrum (at a coverage of 100) indicates diverged homoeologs or cytological diploidization (driven by high aabb in case of polyploid origin). **c,d** Read-based profiling of the genomes of *Clusia major* (left), *C. minor* s.l. (middle), *C. rosea* (right) using GenomeScope (c) and Smudgeplot (d). Based on k-mer frequencies, all species show diploidized genomes of polyploid origin (e.g. AB > AABB). *C. major*: diploidized tetraploid (functional pseudo-diploid of tetraploid origin), *C. minor* s.l.: pseudo-triploid of hexaploid origin, *C. rosea*: pseudo-tetraploid of octoploid origin. Using error corrected long read data could result in an underestimation of heterozygotes states. But based on the author's information, we meet the criteria to use long-reads (distinct genomic peaks and separation from sequencing errors). Although, read coverage of *C. rosea* is too low for an accurate haplotype resolution.

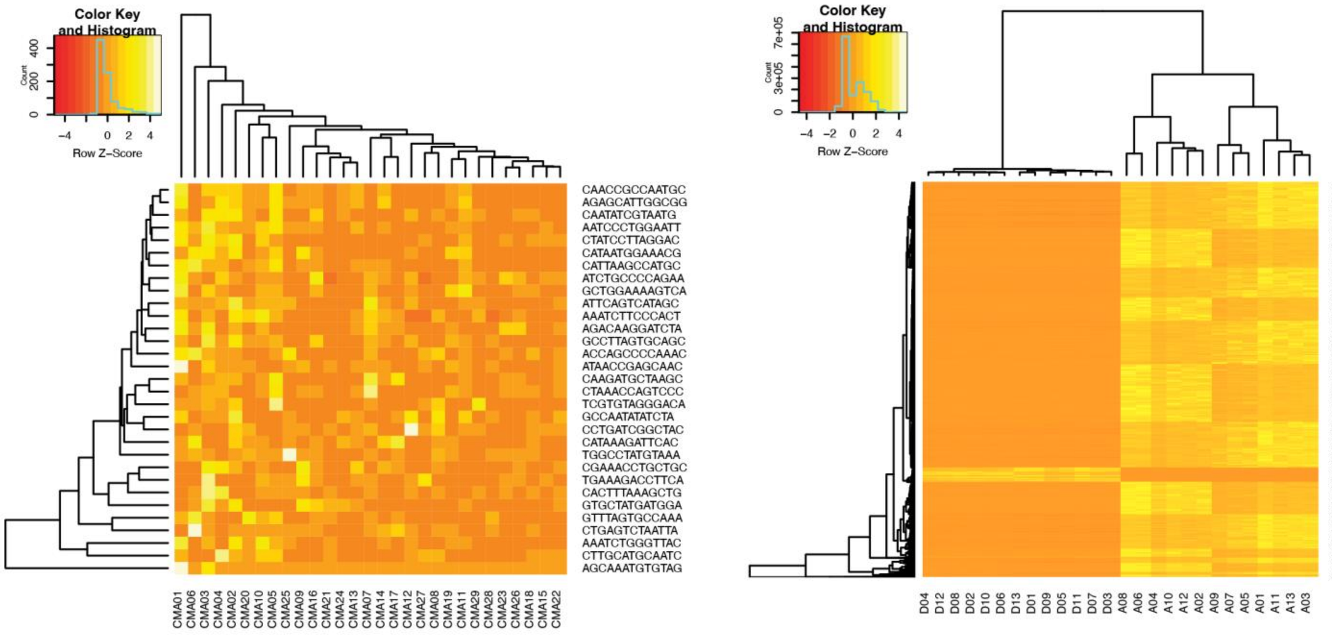

Supplementary Figure 4 | Transposon-mediated subgenome separation of *Clusia major* (left) and Cotton (right). Kmer-based analysis of remnants (e.g. transposons) from hypothetically diverged progenitors prior to an hybridization/alloploidyzation event show no statistically significant clustering of putative subgenomes in *C. major*. The syntenic duplicates of each chromosome (homoeologs) were used as input to search and distinguish between patterns/transposon signatures. A positive control was performed for the well-known allotetraploid cotton (*Gossypium hirsutum*). Altough, transposable elements (TE) most often spread after polyploidyzation and invade both subgenomes. If *C. major* is an allotetraploid (which is not known), this would lead to the assumption that either the two progenitors are so closely related that they have not had time to develop distinct TE complements or, more likely, hybridization occurred long time ago so that subsequent mutations and/or genomic turnover has obscured or erased the evidence for parental genome-specific TE activity like shown in the ancient allohexaploid *Brassica rapa*.

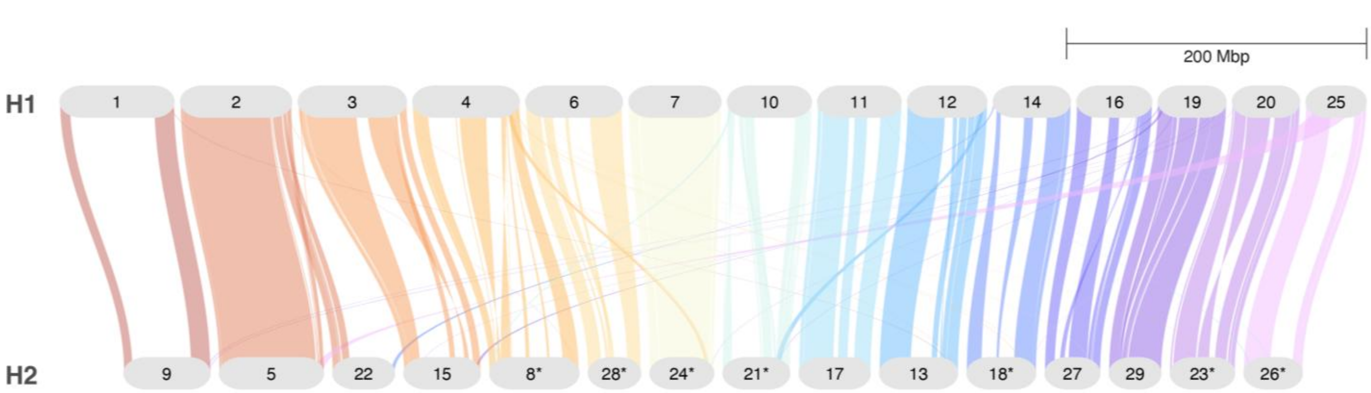

Supplementary Figure 5 | Riparian plot of homoeologous chromosomes in *C. major*. The riparian plot shows macrosyntenic relationships between the groups H1 vs. H2. Pseudo-chromosomes are scaled by physical positions (Mb). Inverted chromosomes are marked with an asterisk*. Proximal accumulation (or elimination) of repetitive elements appears to strongly differentiate homoeologous chromosome pairs by size, such as between the chromosomes 3/15, 6/28, 7/24, 16/27, or 19/29.

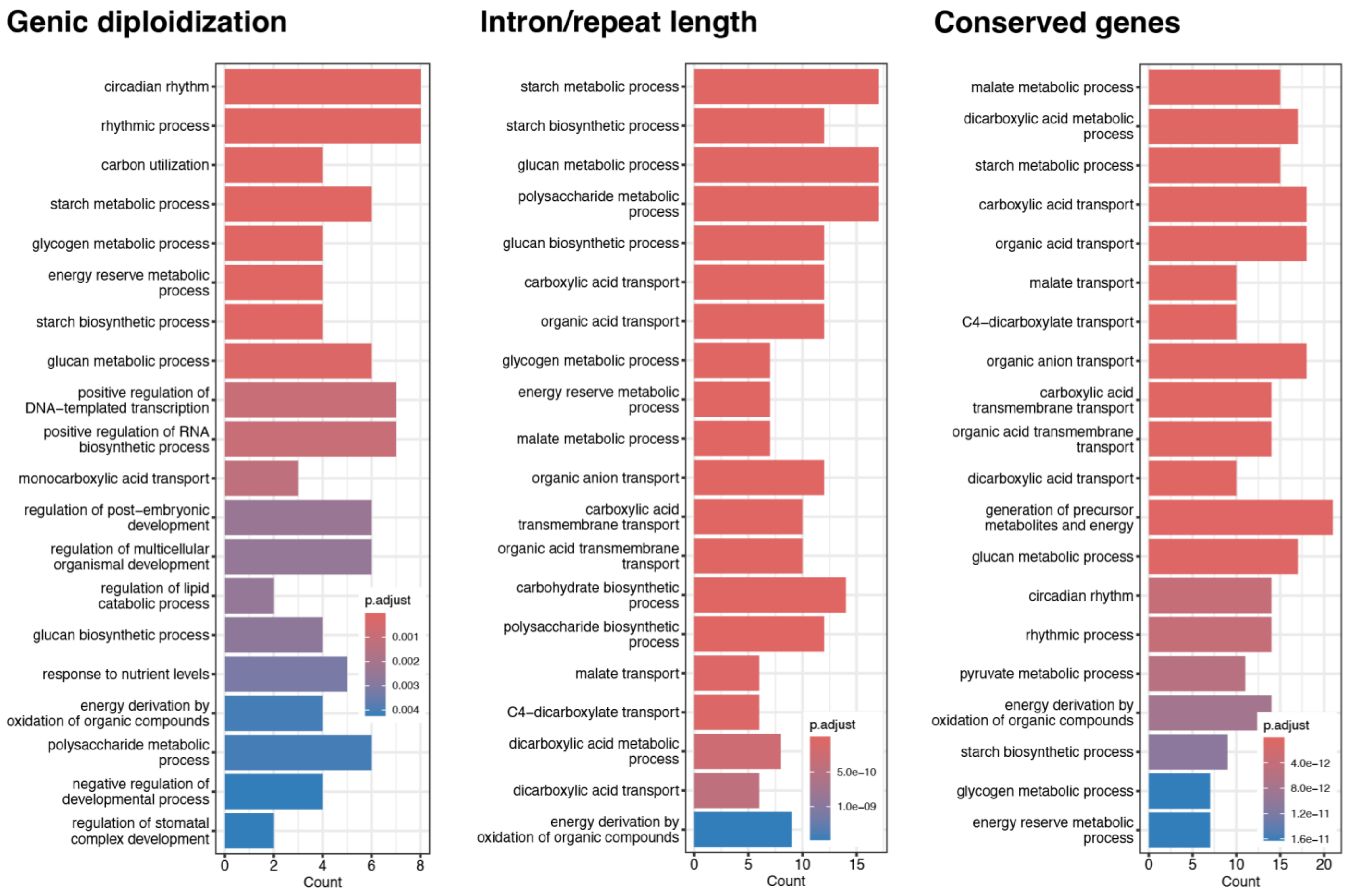

Supplementary Figure 6 | GO term enrichment analysis within CAM-related gene families of *C. major*. For each enrichment (panel), the top 20 categories of statistically over-represented GO terms are shown based on the orthologs and background model of *Arabidopsis thaliana*. Genic diploidization (left) includes gene families with fractionated genes (protein coverage <= 50) or evidence of pseudogenization (premature stop codons, frameshifts, polyA-tails). The panel of intron/repeat length (middle) shows large introns or intronic repeats (minimum genome-wide z-score of 1). The conserved genes (right) represent the CAM-related families comprising genes that show none of these signals (functional gene models from a genomic perspective). While malate and dicarboxylic acid metabolic processes (e.g. *MDH*, *ME*) are more conserved, circadian ryhthm (e.g. *CCA*, *GATA*) and starch/glucan/polysaccharide metabolism are among the GO terms most significantly enriched for genic diploidization or large introns/repeats. Certain gene families contribute to multiple enrichments (panels) as there are variations in the gene copies involved. For instance, conserved and affected homoeologs within the same family (e.g. *AGPase*, *SS*) contributing in all three enrichments to starch metabolic process but the genes of *DPE*, *GWD* and *PGMP* add to the affected panels only. Source data are provided as a Source Data file.

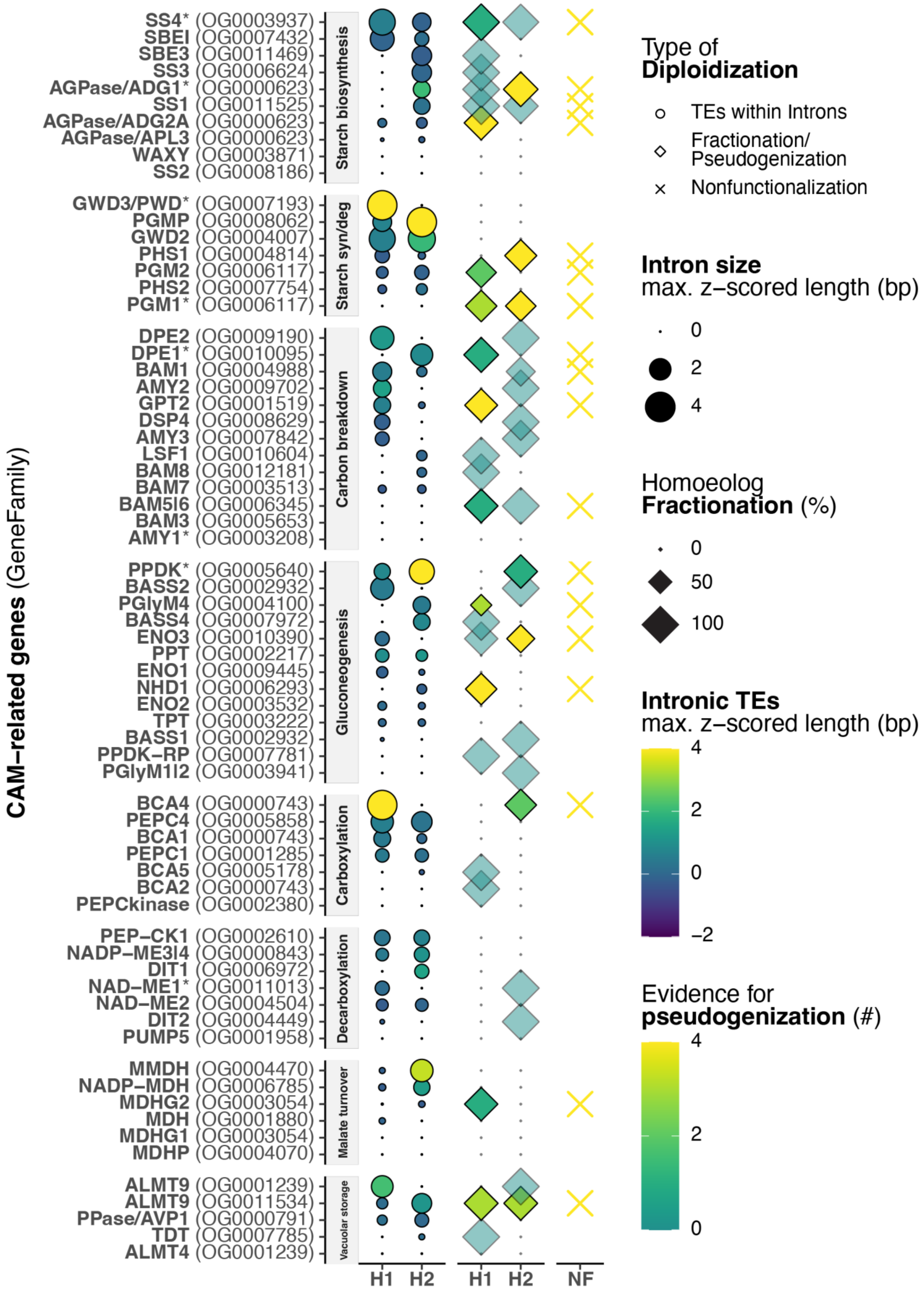

Supplementary Figure 7 | Intronic TEs and homoeolog fractionation affect plastidic CAM and starch degradation pathways in *Clusia major*. Signals of diploidization in CAM-related gene families grouped into specific pathways (Y-axis). Individual gene copies within H1/H2 are summarized to their max. intron/repeat length (z-scored bp) and mean percent of fractionation compared to the corresponding parent/homoeolog (X-axis). Gene families labeled with an asterisk are non-positional (non-syntenic) homoeologs. Data are sorted by intron size. Source data are provided as Supplementary Data 7 and as a Source Data file.

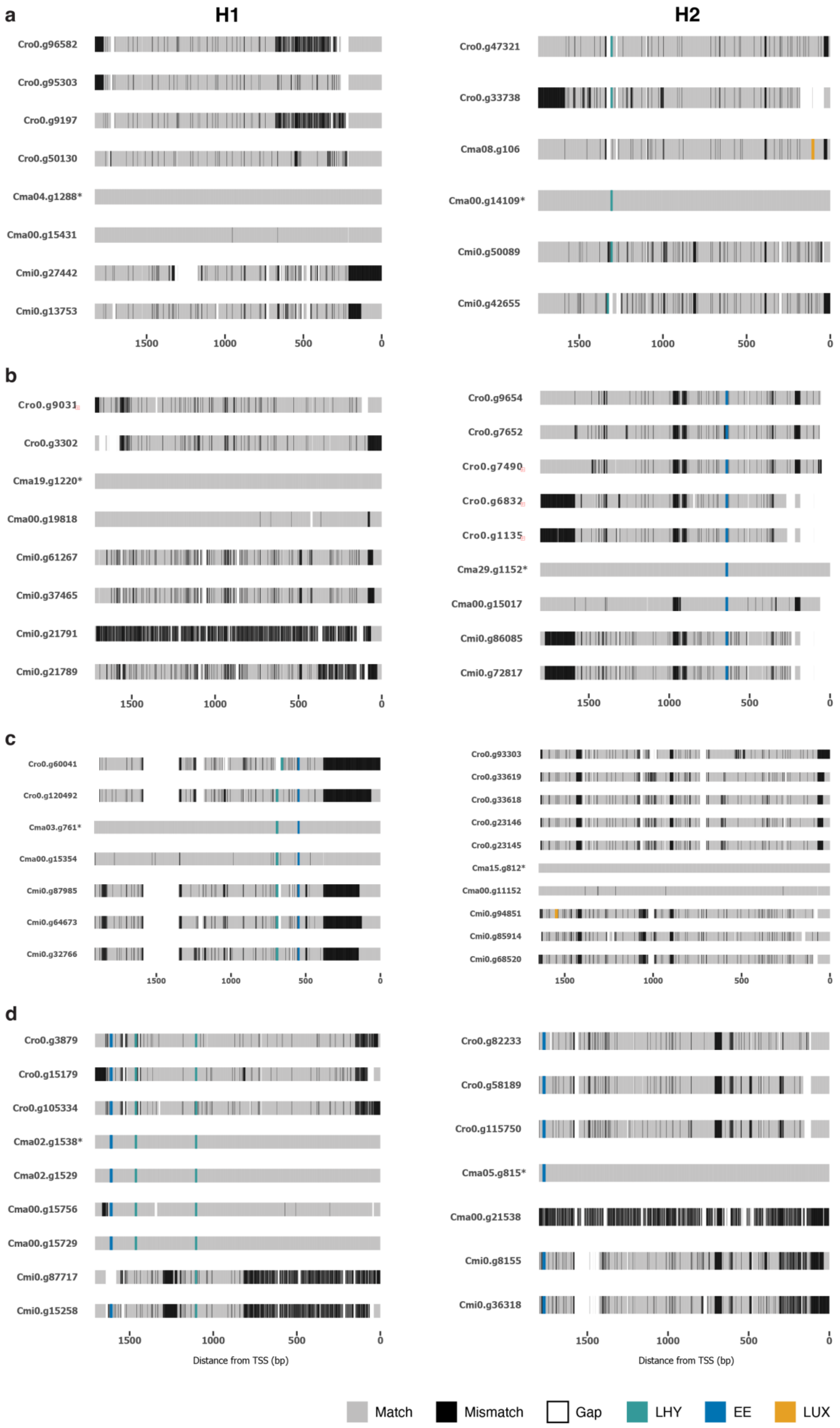

Supplementary Figure 8 | Promoter alignments and *cis*-regulatory motifs of selected CAM-related gene families in *Clusia* spp. The upstream region from the transcriptional start site (TSS) of genes including *cis*-regulatory motifs are shown and separated into the homo(eo)logous groups H1 (left) and H2 (right). Genes marked with an asterisk* were set as reference for the group-wise alignments. The respective species as such can be identified by their gene abbreviations (Cma: *C. major*, Cmi: *C. minor* s.l., Cro: *C. rosea*). Motif presence/absence across groups indicate common sub-functionalization of duplicated genes within the selected gene families. **a** Plastidic phosphoglucomutase (*PGMP*). **b** Phosphoenolpyruvate carboxylase 4 (*PEPC4*). **c** PEPC kinase (*PPCK*). **d** Cytosolic alpha-glucan phosphorylase 2 (*PHS2*/*PHO1*).

CONTROL

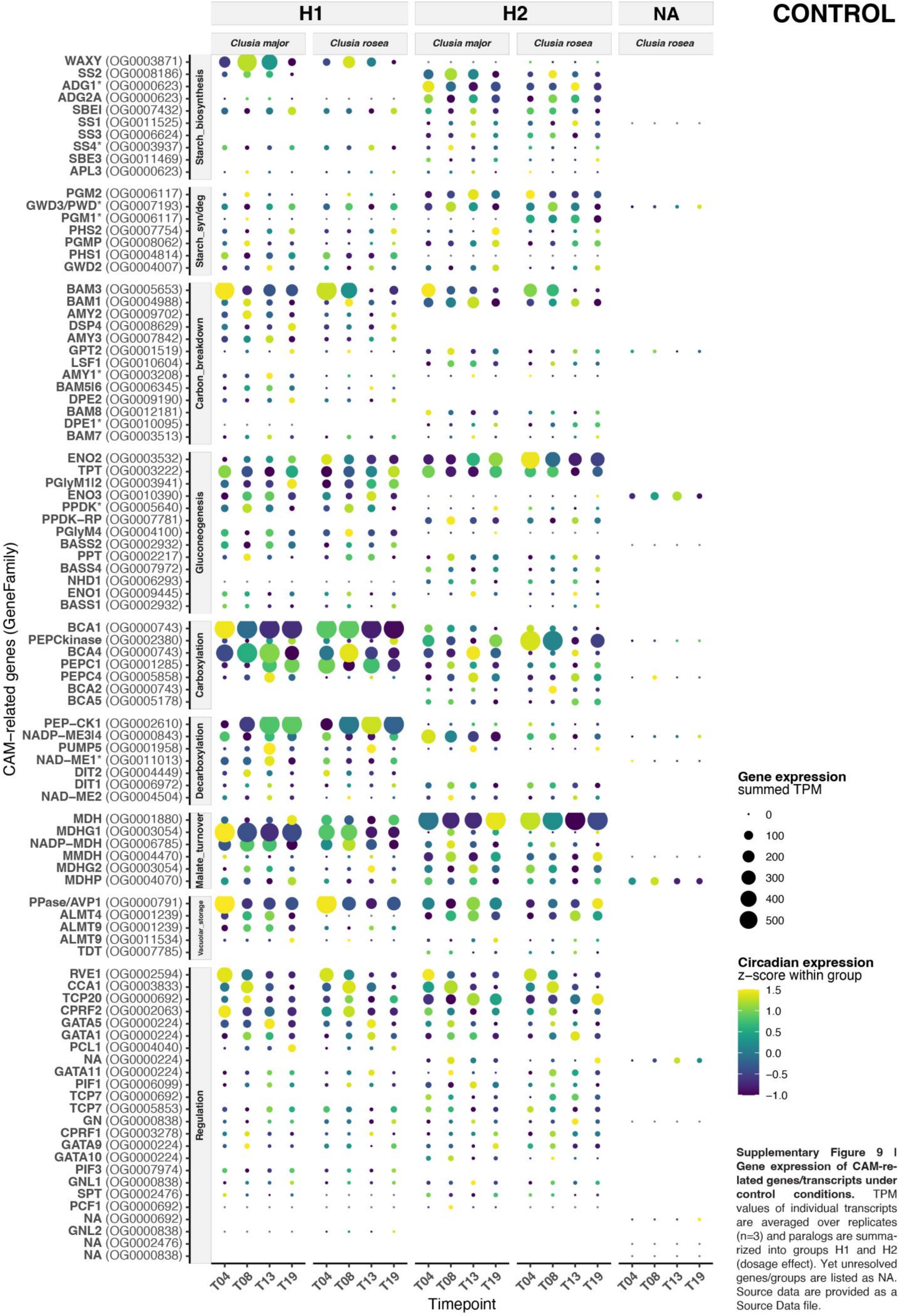

Supplementary Figure 9 | Gene expression of CAM-related genes/transcripts under control conditions. TPM values of individual transcripts are averaged over replicates (n=3) and paralogs are summarized into groups H1 and H2 (dosage effect). Yet unresolved genes/groups are listed as NA. Source data are provided as a Source Data file.

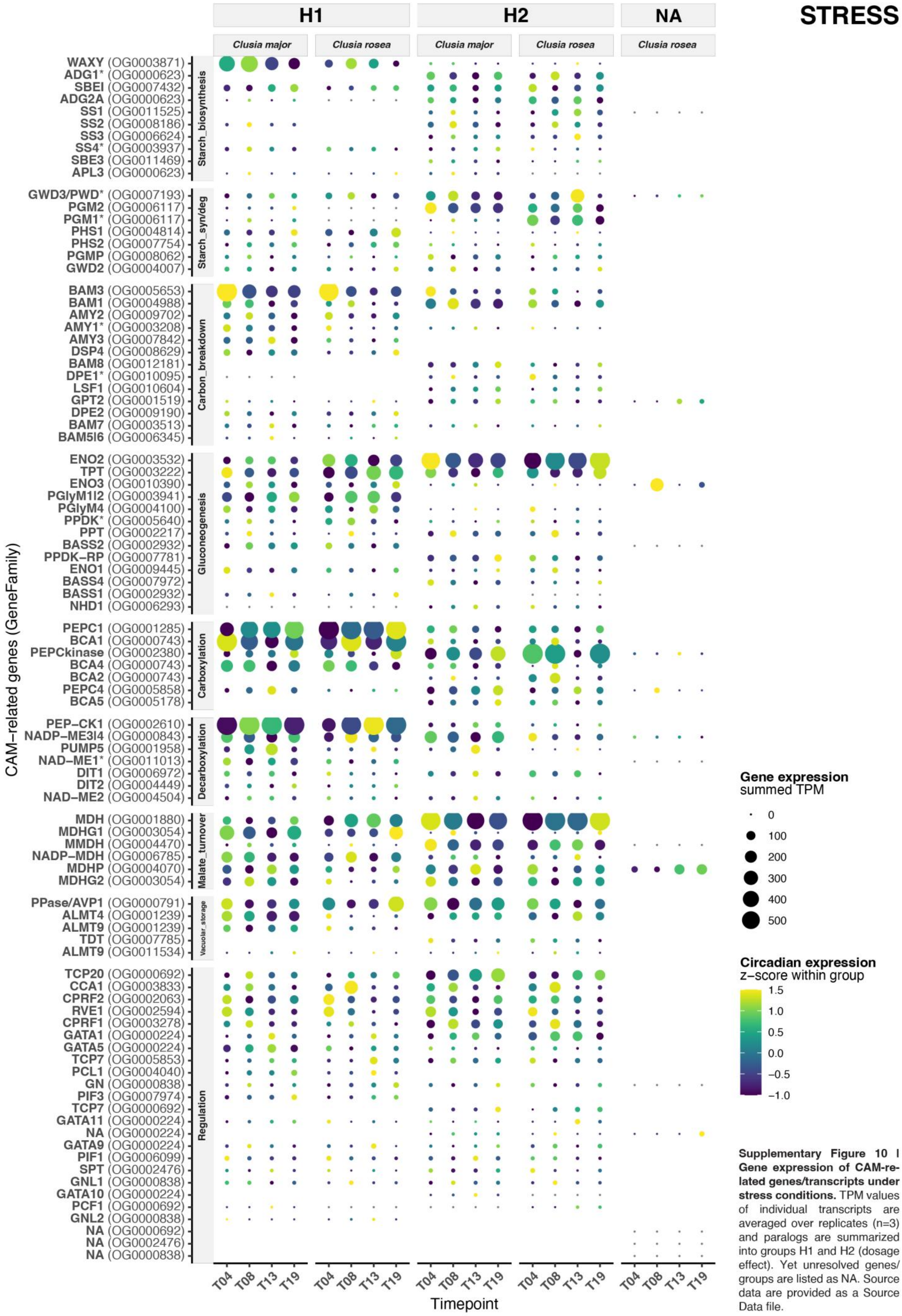

Supplementary Figure 10 I Gene expression of CAM-related genes/transcripts under stress conditions. TPM values of individual transcripts are averaged over replicates (n=3) and paralogs are summarized into groups H1 and H2 (dosage effect). Yet unresolved genes/ groups are listed as NA. Source data are provided as a Source Data file.

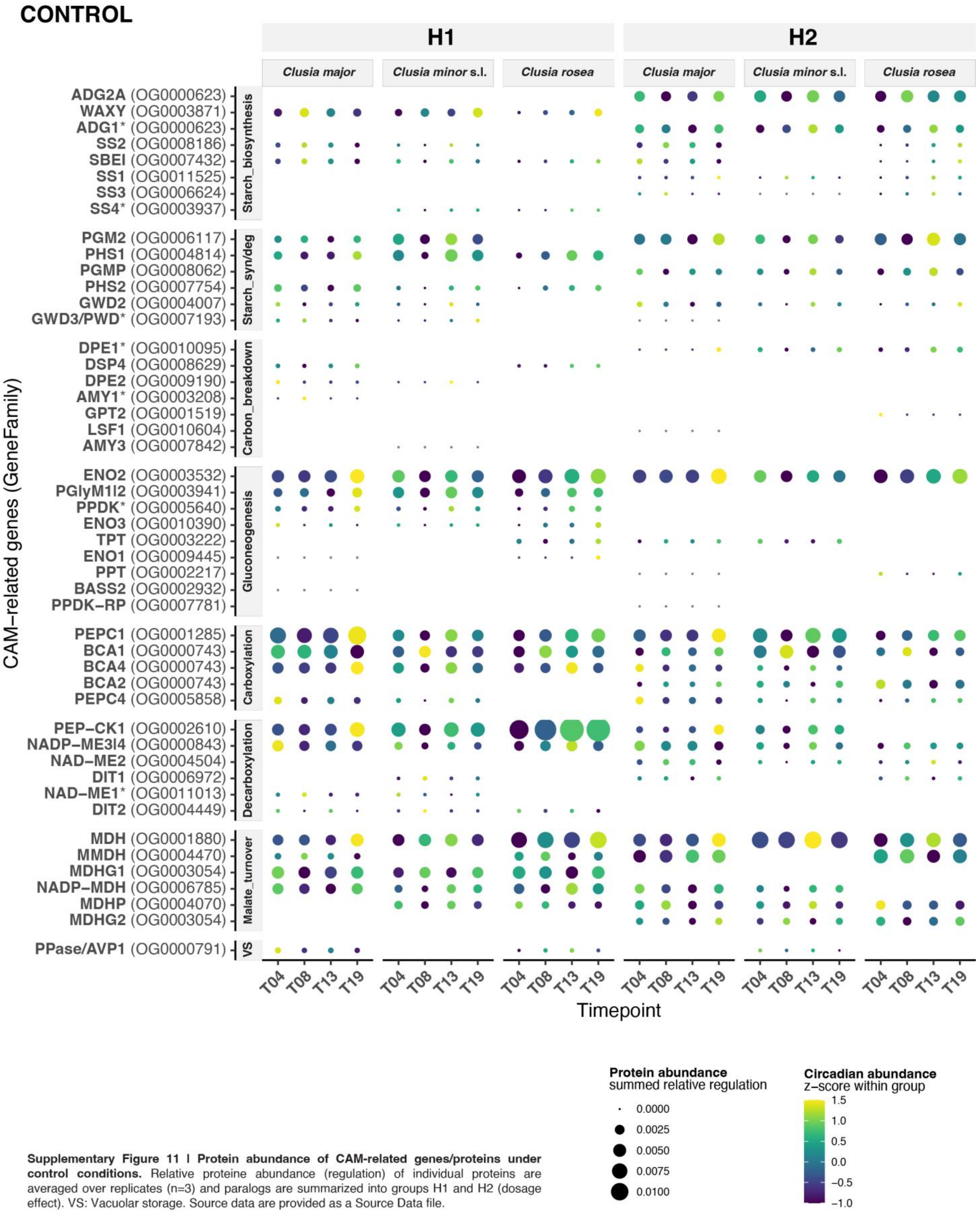

Supplementary Figure 11 | Protein abundance of CAM-related genes/proteins under control conditions. Relative proteine abundance (regulation) of individual proteins are averaged over replicates (n=3) and paralogs are summarized into groups H1 and H2 (dosage effect). VS: Vacuolar storage. Source data are provided as a Source Data file.

STRESS

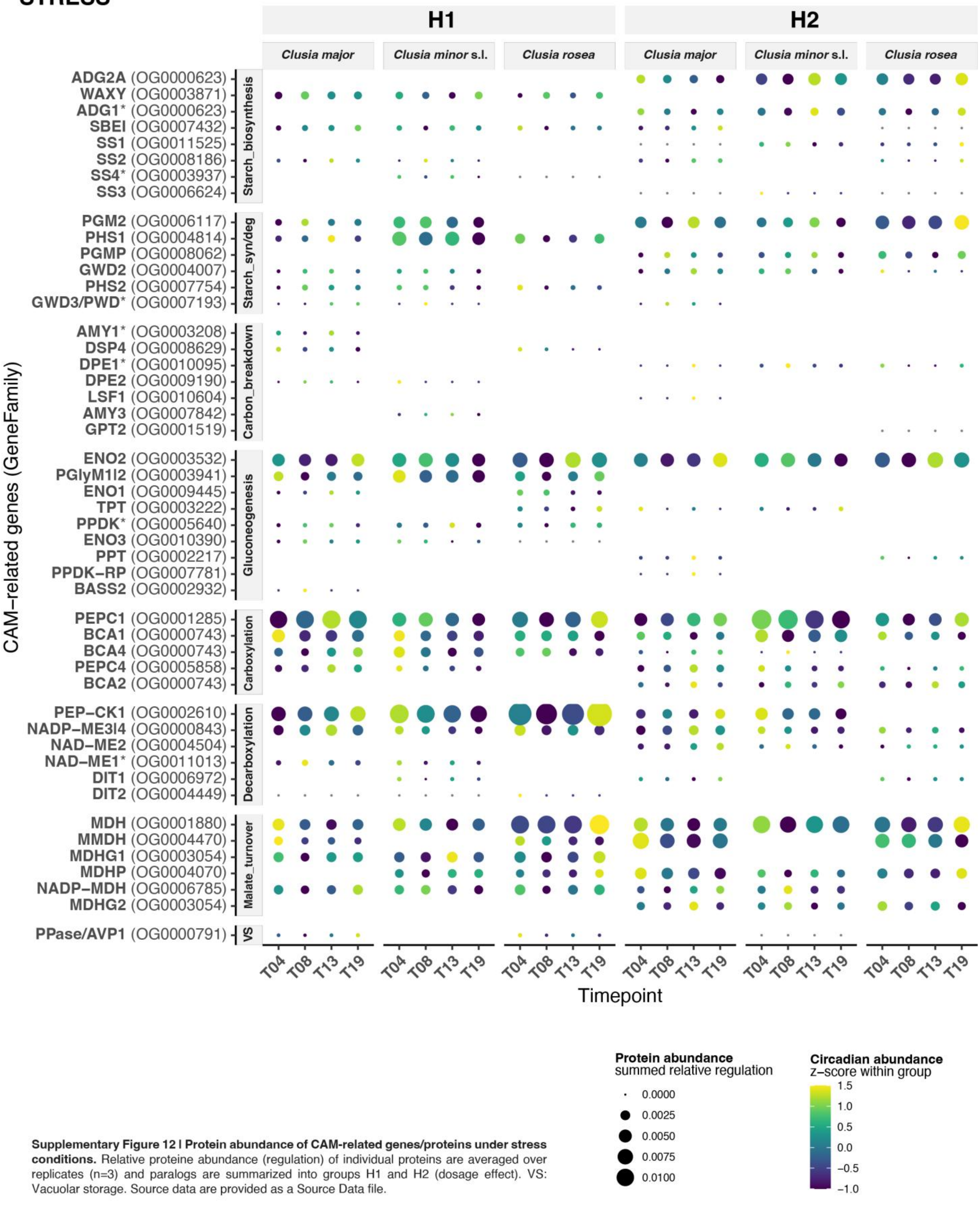

Supplementary Figure 12 | Protein abundance of CAM-related genes/proteins under stress conditions. Relative proteine abundance (regulation) of individual proteins are averaged over replicates (n=3) and paralogs are summarized into groups H1 and H2 (dosage effect). VS: Vacuolar storage. Source data are provided as a Source Data file.

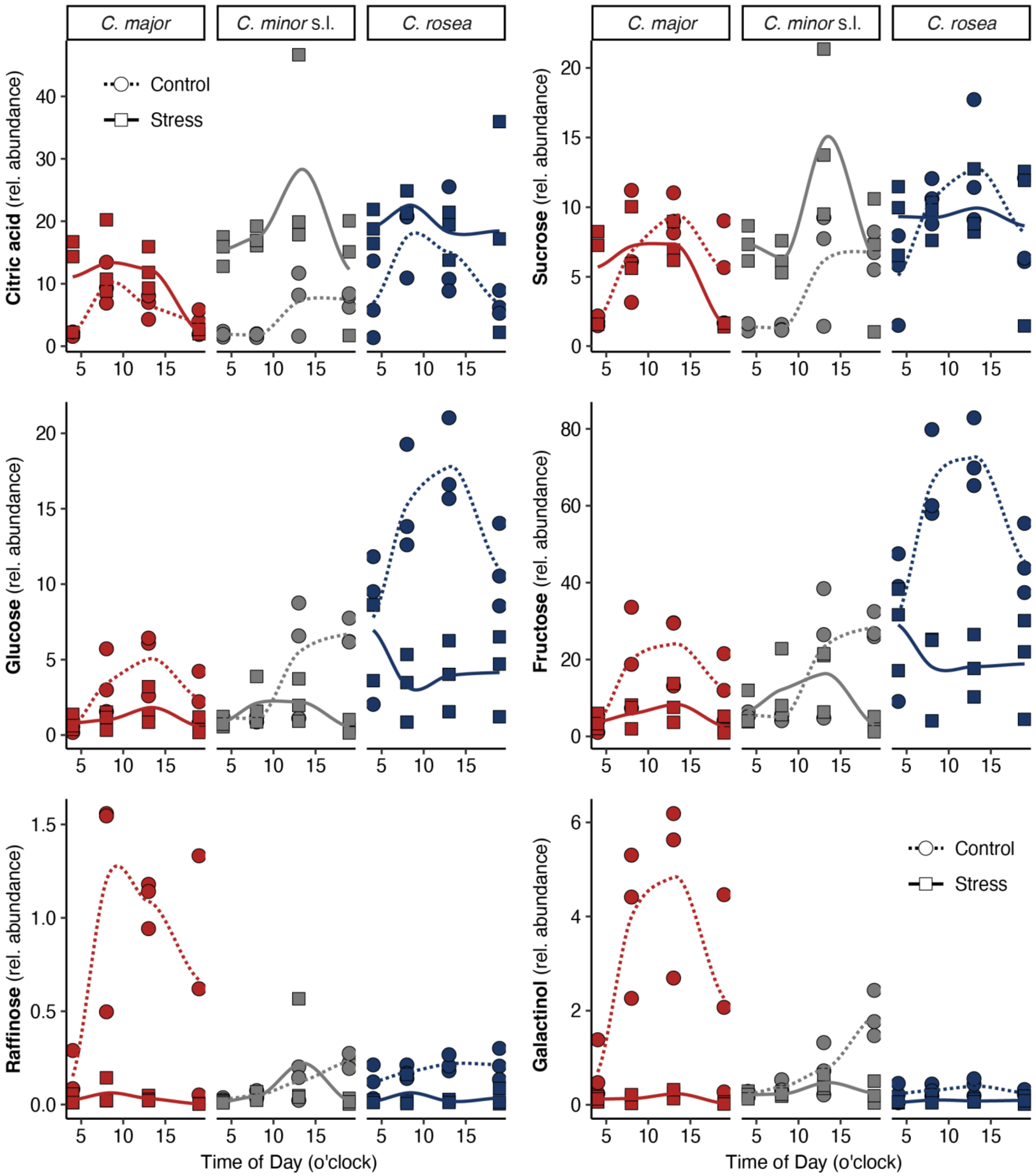

Supplementary Figure 13 | Composed figure of CAM-relevant metabolites in *Clusia* spp. Relative abundance of selected metabolites are shown across a day/night cycle at four timepoints of species/individuals grown under control and stress conditions (n=3). Fluctuations of citric acid suggest it as an alternative to malic acid for nocturnal CO_2 assimilation. Species-dependent diurnal oscillations indicate variable soluble sugars (Glucose, Fructose, Sucrose) are utilized as carbohydrate storage and PEP supply. *C. major* shows contrasting accumulation of other compatible solutes (Raffinose, Galactinol).

a Intra-specific synteny and age distribution of paralogs

gene rank order

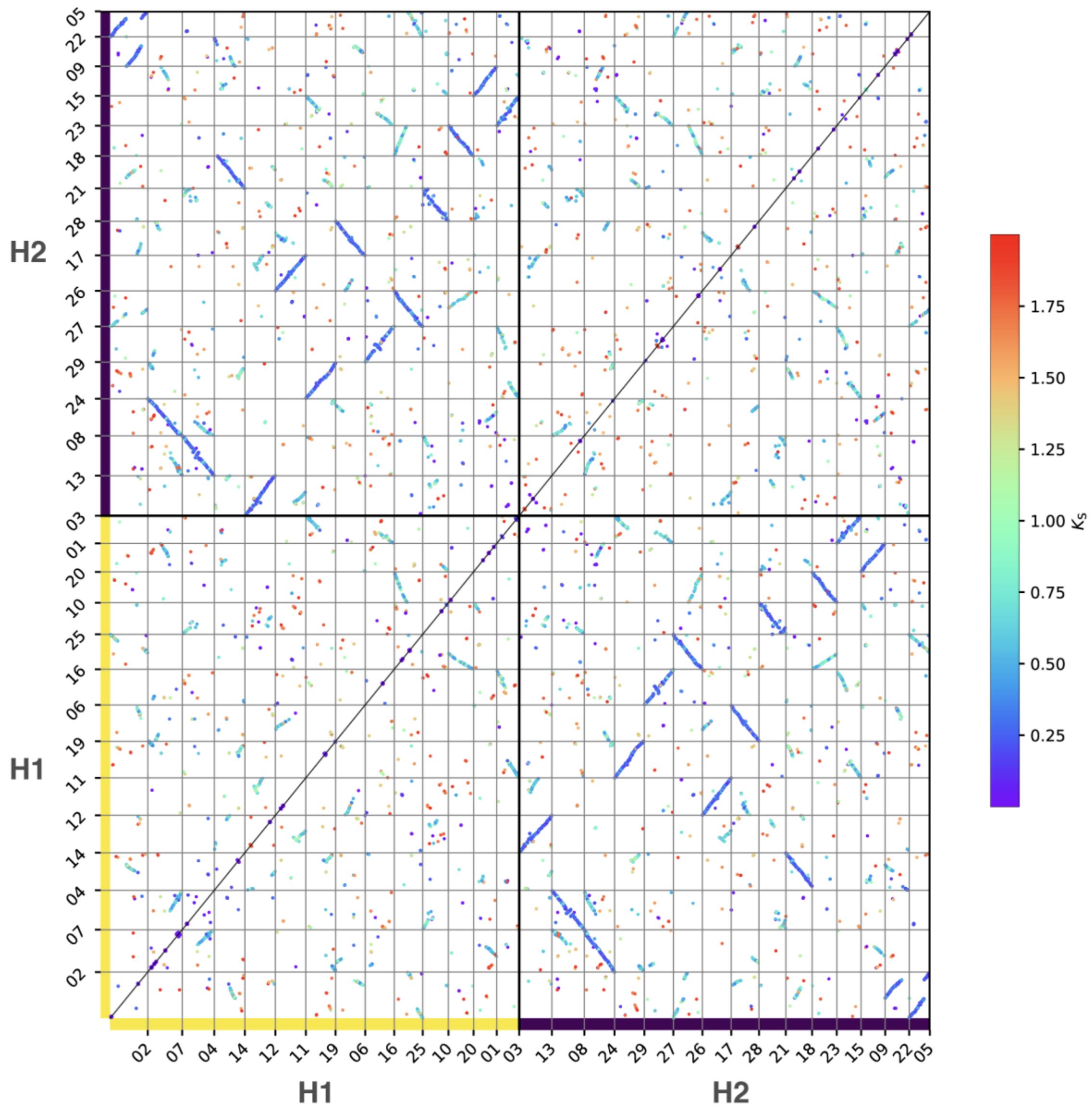

b Relative timing of speciation events and phylogenetic placement of WGDs

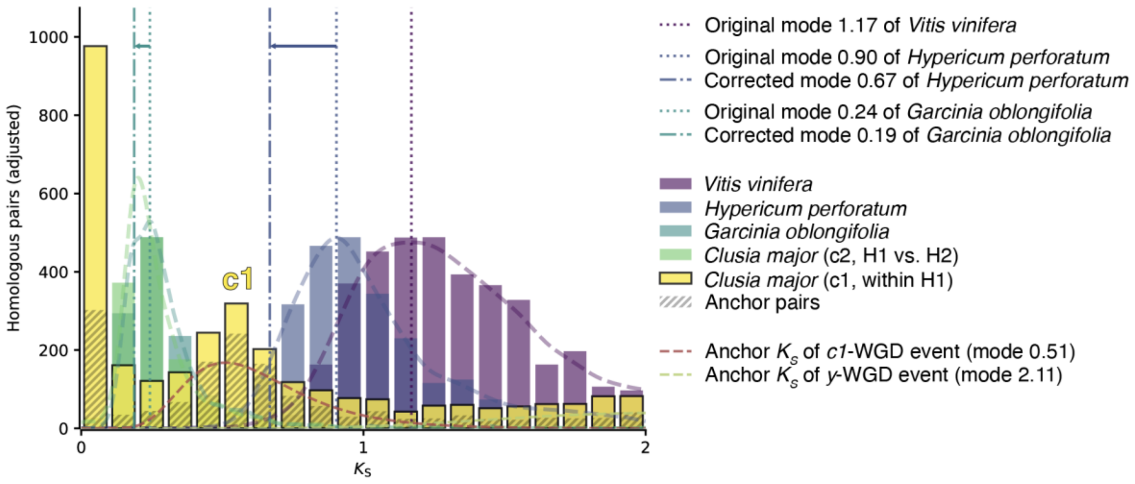

Supplementary Figure 14 | Synteny-based WGD analysis and K_s age distributions. a Dotplot of syntenic paralogs within the primary assembly of *Clusia major*. X/Y unit = gene rank order. K_s cutoff at 2.0 (clade of clusioids). Always four sets of collinear regions/chromosomes (e.g., chr. 23 to 20/25/26) clearly shows another event of polyploidization (former octoploidy). Based on K_s anchor pairs (synonymous substitutions in gene coding regions of syntenic paralogs), the segments split into an older (greenish, c1) and a younger (blue, c2, H1/H2) WGD/WGT event. **b** Relative timing of speciation events and phylogenetic placement of WGDs after rate correction (variation in synonymous substitution rates). Putative subgenomes of *C. major* treated as distinct species. H1 as focal species in comparison to H2 and other clusioids. *Vitis vinifera* as outgroup. Orthologous peaks of distant relatives adjusted/increased for visualization purposes. The c1-WGD event (syntenic paralogs within H1, peak at 0.51) is likely independent of Hypericaceae (speciation of *Hypericum perforatum* at 0.67) but shared within Clusiaceae (at least *Garcinia* and *Clusia*). The divergence of subgenomes (c2) falls in temporal relation to the speciation of *Garcinia oblongifolia*.

Absolute phylogenetic dating of WGDs

Older WGD event (c1)

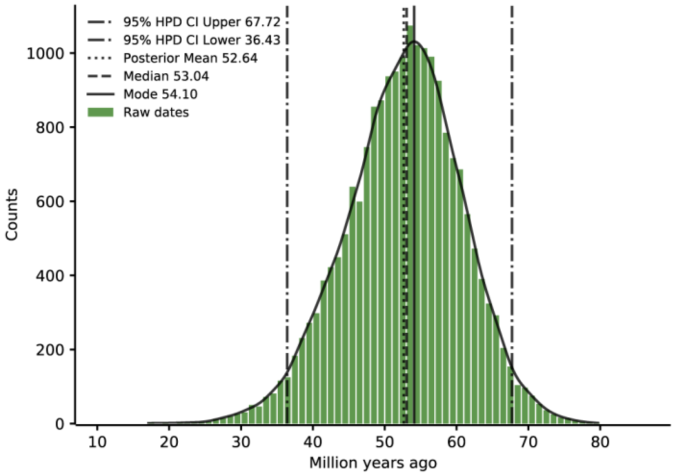

Younger WGD event (c2)

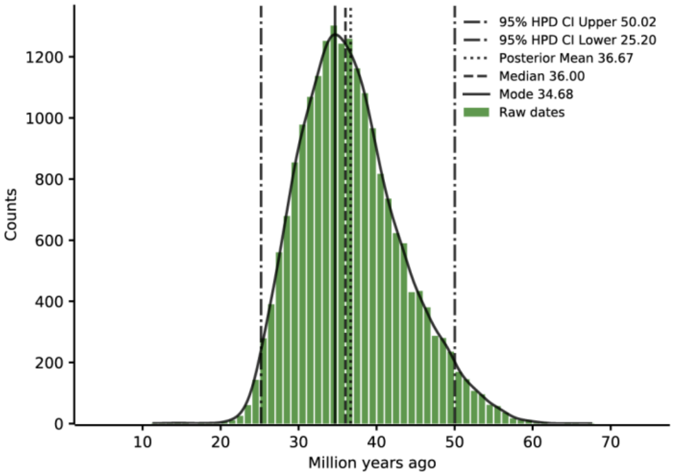

Species tree showing molecular dating of the younger c2-WGD event along speciation/divergence of selected Malpighiales

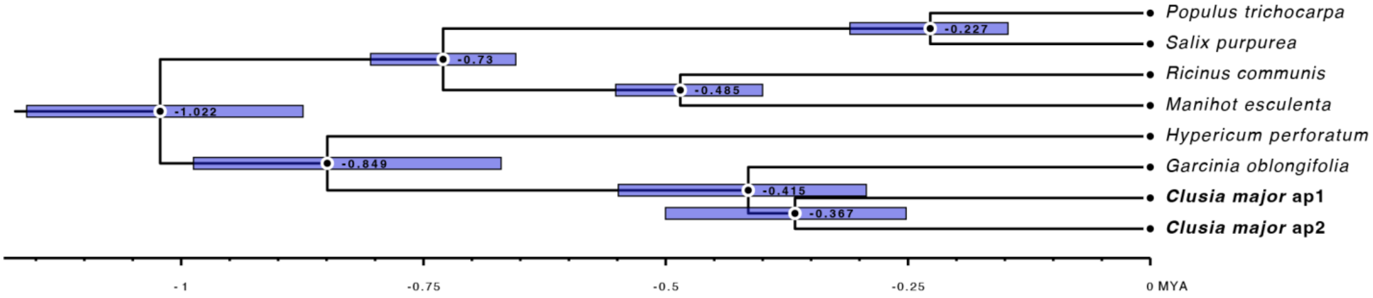

Supplementary Figure 15 | Absolute phylogenetic dating of WGDs. Absolute molecular dating of both WGD events based on reliable anchor pairs (WGD-retained gene duplicates) in *C. major* and orthologoues of selected Malpighiales. Despite highly conserved collinearity at gene dense regions (Supplementary Fig. 12a), both events of polyploidization appear to be ancient and allowing for two separate cycles of diploidization under different atmospheric/climatic conditions. Phylogenomic studies are scarce but would fit the assumption that the c2-WGD event (posterior mean at 36.7 mya) coincides with the radiation of the genus *Clusia*.

Supplementary Table 1. Herbarium vouchers and GenBank accessions.

Species rev.	Species orig.	Specimen	Voucher	Origin	GenBank	Source
Chrysochlamys membranacea Planch. & Triana		*M. Gustafsson 310*	CAY167054, NY	French Guiana	AY145209	Gustafsson and Bittrich [22]
Clusia aripoensis Britton		*A. Borland s.n.*	-	Trinidad (cult.)	AY145197	Gustafsson and Bittrich [22]
Clusia insignis Mart.		*V. Bittrich & M. Amaral s.n.*	UEC	AM, Brazil	AY145167	Gustafsson and Bittrich [22]
Clusia major L.	*C. multiflora*	*A. Berger s.n.*	W0391620, W0356417	Vienna (cult.)	PX225522	This study
Clusia major L.	*C. venosa*	*A. Berger s.n.*	W0356384	Vienna (cult.)	PX225525	This study
Clusia cf. *major*	*C. venosa*	-	-	Darmstadt (cult.)	AJ414726	Vaasen *et al.*[26]
Clusia major L.	*C. rosea*	*L. Hahn 150*	W0356736	Martinique	-	Herbarium W, NHM Vienna
Clusia major L.		*M. Gustafsson 396*	AAU	Martinique	AY145222	Gustafsson and Bittrich [22]
Clusia minor L. s.l.		*M. Gustafsson 291*	NY1204862	cult.	AY145169	Gustafsson and Bittrich [22]
Clusia minor L. s.str.		*B. Hammel 26672*	CR	Costa Rica	MG779428	Luján [6]
Clusia minor L. s.str.		*M. Luján et al. 597*	PMA107340, SCZ	Panama	MF871675	Luján *et al.* [14]
Clusia minor L. s.l.		*A. Berger s.n.*	W0391623, W0356410	Vienna (cult.)	PX225523	This study
Clusia minor L. s.l.		*R. Callejas et al. 4882*	W0391747	Colombia	-	Herbarium W, NHM Vienna
Clusia pratensis Seem.		*B. Hammel 26673*	CR	Costa Rica	MG779426	Luján, 2019[6]
Clusia reginae Paolini-Ruiz		*M. Luján & G. Ghiselli 464*	MERC, MER	Venezuela	MF871671	Luján *et al.* [9], Luján *et al.* [9]
Clusia rosea Jacq.		*A. Berger s.n.*	W0356382	Vienna (cult.)	PX225524	This study
Clusia rosea Jacq.	*C. major*	*A. Berger s.n.*	W0356387	Vienna (cult.)	-	This study
Clusia cf. *rosea*	*C. major*	-	-	Darmstadt (cult.)	AJ414717	Vaasen *et al.* [26]
Clusia rosea Jacq.		-	-	Darmstadt (cult.)	AJ414722	Vaasen *et al.* [26]
Clusia rosea Jacq.		*C. Wright 50*	W0391745	Cuba	-	Herbarium W, NHM Vienna
Clusia rosiflora Planch. & Linden		*M. Luján et al. 467*	MER	Venezuela	MF871687	Luján *et al.* [14]
Clusia viscida Engl.		*M. Gustafsson et al. 507*	AAU	Ecuador	AY145196	Gustafsson and Bittrich [22]
Tovomita longifolia (Rich.) Hochr.	sp.	*S. Mori et al. 24700*	CAY050672, NY	French Guiana	AY145214	Gustafsson and Bittrich [22]

Supplementary Table 2. Genome size estimations (flow cytometry).

Taxon	Date of measurement	pg/1C	S.D.	CV%	Genome size [Gbp]
Clusia major	10.04.19	**1,6825**	0,0027	0,1606	1,65
Clusia minor s.l.	09.04.19	**3,0853**	0,0102	0,3291	3,02
Clusia rosea	09.04.19	**3,2267**	0,0083	0,2576	3,16
Clusia rosea	10.04.19	**3,2165**	0,0048	0,1479	3,15

Supplementary Table 3. Growth/treatment conditions for greenhouse and physiological experiments.

Experiment	Year	Location	Treatment	Species	Day/Night Temp [°C]	Delta Temp [°C]	Day/Night RH [%]	Day/Night VPD [kPa]	Soil H2O [Vol. %]	Photo-period [h]	PAR (noon) [PPFD]	DLI [mol $m^{-2}d^{-1}$]
Literature												
Franco *et al.* [29]			well-watered	C. venosa (aka C. major), C. minor, C. major (aka C. rosea)	26/20	6	58/76	1,83/0,71	-	12	250-300	-
Borland *et al.* [30]			H2O withheld for 10d	C. aripoensis, C. minor, C. rosea	27/19	8	60/80	-	-	11	530	-
Zambrano *et al.* [20]			H2O withheld for 9d	C. minor, C. rosea, C. hilariana, ...	27/18	9	60/75	-	-	12	300	-
Cultivation												
UZA1	until 2021	Greenhouse (UZA1)	-		25/20	5	50/60	1,40/0,79	20	13-16	150	5
UBB	from 2021	Greenhouse (UBB)	-		28/22	6	60/75	1,30/0,50	20	13-16	250	10
Experiments												
OpenGreenhouse (multiomics)	2021 (Q2)	Rooftop (UZA1)	shadow, well-watered	C. major, C. minor s.l. (w/o RNA), C.rosea	27/20	7	42/65	1,86/0,82	>25	15	50-100	3
			exposed, H2O withheld for 7d	C. major, C. minor s.l. (w/o RNA), C.rosea	27/19	8	42/68	2,28/0,70	<10	15	600	30
GasExchange (CO2/GH2O)	2025 (Q2)	Fytochamber3 (UBB)	H2O limited at 10% soil volume	C. major, C.rosea	28/20	8	40/65	2,05/0,68	10	15	300	11
Titratable Acidity (TA)	2025 (Q4)	Fytochamber3 (UBB)	H2O limited at 10% soil volume	C. major, C. minor s.l., C.rosea	28/20	8	40/65	2,05/0,68	10	15	300	11
			well-watered (25-30% soil water vol.)	C. major, C. minor s.l., C.rosea	28/20	8	40/65	2,05/0,68	>25	15	300	11

Supplementary Table 4. Programmatic generation of figures and tables (from raw to source data).

Figure	Raw data (INPUT)	Code file(s)	Data frame (ClusiaDB)	Source data (output)
Figure 1a	Supplementary Table 1	comparative_genomics/phylogeny/tree.sh	-	-
Figure 1b	Dataset 9	ClusiaDB.experiments.R	phenotyping.gasexchange	Fig1b.GasExchange.tsv
Figure 1c	Dataset 9	ClusiaDB.experiments.R	phenotyping.acidity.delta<br>phenotyping.acidity.delta.aov	Fig1c.DeltaTA.tsv<br>Fig1c.ANOVA.tsv
Figure 2	Dataset 2-6	ClusiaDB.circlize.R	-	-
Figure 3a	Dataset 6	ClusiaDB.synteny.R	-	-
Figure 3b	Dataset 4	ClusiaDB.repeats.R	feature.repeats.landscape	Fig3b.RepeatLandscape.tsv
Figure 3c	Dataset 5	ClusiaDB.pseudogenes.R	feature.pseudogenes	Fig3c.ListOfPseudogenes.tsv
Figure 3d	Dataset 6	ClusiaDB.counts.R	feature.counts.og.vitis	Fig3d.GeneFamilies.tsv
Figure 4a	Dataset 2-6	ClusiaDB.diploidization.R	data.diploidization.plot	Fig4a.SummarizedDiploidization.tsv
Figure 4c	Dataset 4	ClusiaDB.diploidization.R	data.diploidization	Fig4c.GenewiseDiploidization.tsv
Figure 5a	Dataset 9	ClusiaDB.experiments.R	opengreenhouse.arduino.soil	Fig5a.SoilWater.tsv
Figure 5b,c	Dataset 9	ClusiaDB.experiments.R	opengreenhouse.photosynq.par	Fig5bc.PAR.tsv
Figure 5d	Dataset 9	ClusiaDB.experiments.R	opengreenhouse.photosynq	Fig5d.PhotosynQ.tsv
Figure 5e,f	Dataset 10	-	-	-
Figure 5g	Dataset 11	ClusiaDB.proteomics.R	data.proteomics.plot	Fig5g.CarboxylatingProteins.tsv
Figure 5h	Dataset 10	ClusiaDB.transcriptomics.R	data.transcriptomics	Fig5h.PPCK.tsv
Figure 5i	Dataset 11	ClusiaDB.proteomics.R	data.proteomics.plot	Fig5i.DecarboxylatingProteins.tsv
Figure 6a	Dataset 10	ClusiaDB.transcriptomics.R	data.transcriptomics.plot	Fig6a.BAM3.tsv
Figure 6b	Dataset 11	ClusiaDB.proteomics.R	data.proteomics.plot	Fig6b.PHS1.tsv
Figure 6c	Dataset 10	ClusiaDB.transcriptomics.R	data.transcriptomics.plot	Fig6c.PGMP.tsv
Figure 6d	-	-	-	Fig6d.Starch.tsv
Figure 6e,f	Dataset 12	-	-	-
Supplementary Figure 1a	Dataset 9	ClusiaDB.experiments.R	phenotyping.env.water	Supplementary_Fig1a.SoilWater.tsv
Supplementary Figure 1b	Dataset 9	ClusiaDB.experiments.R	phenotyping.env.par	Supplementary_Fig1b.PAR.tsv
Supplementary Figure 1b	Dataset 9	ClusiaDB.experiments.R	phenotyping.env.fytotron	Supplementary_Fig1b.ENV.tsv
Supplementary Figure 1c	Dataset 9	ClusiaDB.experiments.R	phenotyping.acidity	Supplementary_Fig1c.TA.tsv
Supplementary Figure 1d	Dataset 9	ClusiaDB.experiments.R	phenotyping.acidity.delta	Supplementary_Fig1d.DeltaTA.tsv
Supplementary Figure 3	-	assembly/genomescope/genomescope.sh	-	-
Supplementary Figure 4	-	assembly/kbsm/run.sh	-	-
Supplementary Figure 5	Dataset 6	ClusiaDB.synteny.R	-	-
Supplementary Figure 6	Supplementary Data 6	ClusiaDB.enrichment.R	feature.goterms.diploidization	Supplementary_Fig6.GenicDiploidization.tsv
Supplementary Figure 6	Supplementary Data 6	ClusiaDB.enrichment.R	feature.goterms.repeats	Supplementary_Fig6.Repeats.tsv
Supplementary Figure 6	Supplementary Data 6	ClusiaDB.enrichment.R	feature.goterms.conserved	Supplementary_Fig6.ConservedGenes.tsv
Supplementary Figure 7	Dataset 2-6	ClusiaDB.diploidization.R	data.diploidization.plot	Supplementary_Fig7.SummarizedDiploidization.tsv
Supplementary Figure 8	Dataset 10	ClusiaDB.transcriptomics.R	data.transcriptomics.plot	Supplementary_Fig8.GeneExpression.tsv
Supplementary Figure 9	Dataset 11	ClusiaDB.proteomics.R	data.proteomics.plot	Supplementary_Fig9.ProteinAbundance.tsv
Supplementary Figure 10	Dataset 6	-	-	-
Supplementary Figure 11	Dataset 12	-	-	-
Supplementary Figure 12	Dataset 2-3	comparative_genomics/wgd/wgd.*.sh	-	-
Supplementary Figure 13	Dataset 2-3	comparative_genomics/wgd/wgd.dating.sh	-	-

Supplementary references

1. Luján, M., Leverett, A. & Winter, K. Forty years of research into crassulacean acid metabolism in the genus *Clusia*: anatomy, ecophysiology and evolution. *Annals of Botany* **132**, 739–752 (2023).
2. Lüttge, U. *Clusia*: Holy Grail and enigma. *J Exp Bot* **59**, 1503–1514 (2008).
3. D'Arcy, W. G. *Flora of Panama: Checklist and Index*. (Missouri Botanical Garden Press, St. Louis, Mo, 1987).
4. Hammel, B. E. *Manual de plantas de Costa Rica: Clusiaceae-Gunneraceae*. (Missouri Botanical Garden Press, St. Louis, 2010).
5. Herbert-Doctor, L. A. *et al. Clusia suborbicularis* is not a synonym of *Clusia flava*: Molecular and metabolomic evidence. *Taxon* **70**, 1229–1238 (2021).
6. Luján, M. Playing the Taxonomic Cupid: Matching pistillate and staminate conspecifics in dioecious *Clusia* (Clusiaceae). *Systematic Botany* **44**, 548–559 (2019).
7. Alencar, A. C., Nascimento-Jr, J. E. D., Bittrich, V., Farias-Castro, A. S. & Amaral, M. D. C. E. D. Two new species of *Clusia* sect. *Cordylandra* (Clusiaceae) from the *brejo de altitude* vegetation, plateau of Ibiapaba, Ceará, Brazil. *Phytotaxa* **460**, 259–268 (2020).
8. Alencar, A. C., Bittrich, V. & Amaral, M. do C. E. *Clusia nascimentojuniorii*: A new species of sect. Phloianthera from the Amazon (Brazil). *Systematic Botany* **46**, 370–374 (2021).
9. Luján, M., Paolini-Ruiz, J., Sanoja, E., Rojas, C. A. & Ely, F. Integrative taxonomy led to recognising *Clusia reginae* (Clusiaceae), a new tree species from the Venezuelan Andes. *Kew Bull* **79**, 191–200 (2024).
10. Luján, M. *Clusia chuj* (Clusiaceae), a new tree species from the border between Guatemala and Mexico. *Kew Bull* **78**, 533–537 (2023).
11. Luján, M., Wodcke, E. & Hammel, B. Two new species of *Clusia* (Clusiaceae) endemic to Costa Rica. *Kew Bull* **79**, 881–887 (2024).
12. Luján, M., Cacho, N. I., Pérez-Farrera, M. Á. & Hammel, B. *Clusia falcata* (Clusiaceae), an endangered species with exceptionally narrow leaves endemic to Chiapas, Mexico. *Kew Bull* **76**, 645–650 (2021).
13. Wodcke, E. A., Luján, M. & Hammel, B. *Clusia salicifolia* (Clusiaceae), a new hemiepiphyte species from Costa Rica and Panama. *Kew Bull* **79**, 185–190 (2024).

14. Luján, M., Aranda, J., Virgo, A. & Winter, K. *Clusia guabalensis* (Clusiaceae), a new hemiepiphyte species with floral resins from the Atlantic wet forest in Panama. *Brittonia* **70**, 412–417 (2018).
15. Luján, M., Idárraga, Á. & Hammel, B. *Clusia hirsuta*, a new species from *Clusia* sect. Retinostemon and the first description of trichomes in the genus. *Novon, A Journal for Botanical Nomenclature* **26**, 154–158 (2018).
16. Hammel, B. E. New species of Clusiaceae from Central America with notes on *Clusia* and synonymy in the tribe Clusieae. *Selbyana* **9**, 112–120 (1986).
17. Howard, R. A. *Flora of the Lesser Antilles, Leeward and Windward Islands: Vol. 5: Dicotyledoneae-Part 2 by Richard A. Howard & Allan J. Bornstein*. (Arnold Arboretum, Harvard Univ., 1989).
18. Lüttge, U. Photosynthetic flexibility and ecophysiological plasticity: questions and lessons from *Clusia*, the only CAM tree, in the neotropics. *New Phytologist* **171**, 7–25 (2006).
19. Winter, K., Garcia, M. & Holtum, J. A. M. On the nature of facultative and constitutive CAM: environmental and developmental control of CAM expression during early growth of *Clusia*, *Kalanchoë*, and *Opuntia*. *Journal of Experimental Botany* **59**, 1829–1840 (2008).
20. Barrera Zambrano, V. A., Lawson, T., Olmos, E., Fernández-García, N. & Borland, A. M. Leaf anatomical traits which accommodate the facultative engagement of crassulacean acid metabolism in tropical trees of the genus *Clusia*. *J Exp Bot* **65**, 3513–3523 (2014).
21. Bittrich, V. & Stevens, P. F. Lectotypification of the names *Clusia major* and *C. minor* (Clusiaceae). *Taxon* **47**, 117–122 (1998).
22. Gustafsson, M. H. G. & Bittrich, V. Evolution of morphological diversity and resin secretion in flowers of *Clusia* (Clusiaceae): insights from ITS sequence variation. *Nordic Journal of Botany* **22**, 183–203 (2002).
23. Winter, K. & Holtum, J. A. M. Facultative crassulacean acid metabolism (CAM) plants: powerful tools for unravelling the functional elements of CAM photosynthesis. *Journal of Experimental Botany* **65**, 3425–3441 (2014).
24. Borland, A. M., Griffiths, H., Broadmeadow, M. S. J., Fordham, M. C. & Maxwell, C. Short-term changes in carbon-isotope discrimination in the C3-CAM intermediate *Clusia minor* L. growing in Trinidad. *Oecologia* **95**, 444–453 (1993).
25. Haag-Kerwer, A., Franco, A. C. & Lüttge, U. The effect of temperature and light on gas exchange and acid accumulation in the C3-CAM plant *Clusia minor* L. *Journal of Experimental Botany* **43**, 345–352 (1992).

26. Vaasen, A., Begerow, D., Lüttge, U. & Hampp, R. The genus *Clusia* L.: Molecular evidence for independent evolution of photosynthetic flexibility. *Plant Biology* **4**, 86–93 (2002).
27. Howard, R. A. Some Guttiferae of the Lesser Antilles. *Journal of the Arnold Arboretum* **43**, 389–399 (1962).
28. Gustafsson, M. H. G., Winter, K. & Bittrich, V. Diversity, Phylogeny and Classification of *Clusia*. in *Clusia: A Woody Neotropical Genus of Remarkable Plasticity and Diversity* (ed. Lüttge, U.) 95–116 (Springer, Berlin, Heidelberg, 2007). doi:10.1007/978-3-540-37243-1_7.
29. Franco, A. C., Ball, E. & Lüttge, U. Patterns of gas exchange and organic acid oscillations in tropical trees of the genus *Clusia*. *Oecologia* **85**, 108–114 (1990).
30. Borland, A. M., Técsi, L. I., Leegood, R. C. & Walker, R. P. Inducibility of crassulacean acid metabolism (CAM) in *Clusia* species; physiological/biochemical characterisation and intercellular localization of carboxylation and decarboxylation processes in three species which exhibit different degrees of CAM. *Planta* **205**, 342–351 (1998).
31. Lüttge, U. *Clusia: A Woody Neotropical Genus of Remarkable Plasticity and Diversity*. (Springer, Berlin, 2007).
32. Sage, R. F., Gilman, I. S., Smith, J. A. C., Silvera, K. & Edwards, E. J. Atmospheric CO2 decline and the timing of CAM plant evolution. *Annals of Botany* **132**, 753–770 (2023).
33. Vaasen, A., Begerow, D. & Hampp, R. Phosphoenolpyruvate carboxylase genes in C3, crassulacean acid metabolism (CAM) and C3/CAM intermediate species of the genus *Clusia*: rapid reversible C3/CAM switches are based on the C3 housekeeping gene. *Plant, Cell & Environment* **29**, 2113–2123 (2006).
34. Lüttge, U. Photosynthetic flexibility and ecophysiological plasticity: questions and lessons from *Clusia*, the only CAM tree, in the neotropics. *New Phytologist* **171**, 7–25 (2006).
