# Supplementary material for: Clusia genomes shed light on the evolution and diversity of crassulacean acid metabolism physiotypes
Source: Nat Commun. 2026 May 5;17:3937. doi: 10.1038/s41467-026-71958-z (PMC13144421; doi:10.1038/s41467-026-71958-z)
Supplement: Supplementary file 3 — Description of Additional Supplementary Files [file 41467_2026_71958_MOESM3_ESM.pdf]

Description of Additional Supplementary Files

Supplementary Data 1. Sequencing statistics and read coverage

Supplementary Data 2. Assembly performance of draft genomes

Supplementary Data 3. Scaffolding efficiency and HiC contact maps

Supplementary Data 4. Statistics of pseudo-haplotype assembly

Supplementary Data 5. Pseudogenes in the chromosome-level assembly of *C. major*

Supplementary Data 6. Combined data/signals of diploidization for CAM-related gene families

Supplementary Data 7. Transit peptide prediction of genes encoding for *PGMP*

Supplementary Data 8. *Cis*-regulatory motifs in promoters of CAM-related genes

Supplementary Data 9. HMW gDNA extraction
