# Supplementary material for: Clusia genomes shed light on the evolution and diversity of crassulacean acid metabolism physiotypes
Source: Nat Commun. 2026 May 5;17:3937. doi: 10.1038/s41467-026-71958-z (PMC13144421; doi:10.1038/s41467-026-71958-z)
Supplement: Supplementary file 13 — Reporting Summary [file 41467_2026_71958_MOESM13_ESM.pdf]

nature portfolio

Corresponding author(s): Wolfram Weckwerth

Last updated by author(s): Mar 28, 2026

Reporting Summary

Nature Portfolio wishes to improve the reproducibility of the work that we publish. This form provides structure for consistency and transparency in reporting. For further information on Nature Portfolio policies, see our Editorial Policies and the Editorial Policy Checklist.

Statistics

For all statistical analyses, confirm that the following items are present in the figure legend, table legend, main text, or Methods section.

n/a	Confirmed	
☐	☒	The exact sample size (*n*) for each experimental group/condition, given as a discrete number and unit of measurement
☐	☒	A statement on whether measurements were taken from distinct samples or whether the same sample was measured repeatedly
☐	☒	The statistical test(s) used AND whether they are one- or two-sided<br>*Only common tests should be described solely by name; describe more complex techniques in the Methods section.*
☐	☒	A description of all covariates tested
☐	☒	A description of any assumptions or corrections, such as tests of normality and adjustment for multiple comparisons
☐	☒	A full description of the statistical parameters including central tendency (e.g. means) or other basic estimates (e.g. regression coefficient) AND variation (e.g. standard deviation) or associated estimates of uncertainty (e.g. confidence intervals)
☐	☒	For null hypothesis testing, the test statistic (e.g. *F*, *t*, *r*) with confidence intervals, effect sizes, degrees of freedom and *P* value noted<br>*Give P values as exact values whenever suitable.*
☒	☐	For Bayesian analysis, information on the choice of priors and Markov chain Monte Carlo settings
☒	☐	For hierarchical and complex designs, identification of the appropriate level for tests and full reporting of outcomes
☒	☐	Estimates of effect sizes (e.g. Cohen's *d*, Pearson's *r*), indicating how they were calculated

Our web collection on statistics for biologists contains articles on many of the points above.

Software and code

Policy information about availability of computer code

Data collection	see MM
Data analysis	The pipelines, scripts and code files used for analyses in this study have been deposited on figshare [https://doi.org/10.6084/m9.figshare.26212895] and Zenodo [https://doi.org/10.5281/zenodo.19212189], and are available as public repository on GitHub [https://github.com/hanneskramml/Clusia]. A dedicated compute capsule to assess the Clusia panomics database is published on Code Ocean [https://doi.org/10.24433/CO.2105665.v2].

For manuscripts utilizing custom algorithms or software that are central to the research but not yet described in published literature, software must be made available to editors and reviewers. We strongly encourage code deposition in a community repository (e.g. GitHub). See the Nature Portfolio guidelines for submitting code & software for further information.

Data

Policy information about availability of data

All manuscripts must include a data availability statement. This statement should provide the following information, where applicable:

- Accession codes, unique identifiers, or web links for publicly available datasets
- A description of any restrictions on data availability
- For clinical datasets or third party data, please ensure that the statement adheres to our policy

The DNA/RNA sequencing-related data generated in this study have been deposited at NCBI under the umbrella project PRJNA1334428 [https://www.ncbi.nlm.nih.gov/bioproject/PRJNA1334428/]. ITS marker sequences are available in GenBank under the accession numbers PX225522-PX225525 [https://

www.ncbi.nlm.nih.gov/nuccore/?term=PX225522:PX225525[accn]]. gDNA sequencing libraries (PacBio continuous long-reads, Illumina HiC short-reads) are publicly available within the sequence read archive (SRA) under project PRJNA1183765 with the following BioSample:SRA accessions SAMN44635471: SRX26662290, SRX26679105 (C. major); SAMN44635472: SRX26662291 (C. minor s.l.); and SAMN44635473: SRX26662292 (C. rosea) [https://www.ncbi.nlm.nih.gov/sra/?term=PRJNA1183765]. The primary chromosome-level assembly of C. major (predominant/principal pseudo-haplotype) is deposited under the GenBank accession GCA_056099205.1 [https://www.ncbi.nlm.nih.gov/datasets/genome/GCA_056099205.1/]. The genome for the alternative pseudo-haplotype of C. major can be accessed via GCA_056098825.1 [https://www.ncbi.nlm.nih.gov/datasets/genome/GCA_056098825.1/]. The draft genome assemblies of C. minor s.l. and C. rosea (contig-level) are linked under the BioProject number PRJNA1183765 [https://www.ncbi.nlm.nih.gov/bioproject/PRJNA1183765/]. mRNA sequencing data were deposited in SRA under the project accession PRJNA1197736 and in NCBI's Gene Expression Omnibus accessible through GEO Series accession number GSE290226 [https://www.ncbi.nlm.nih.gov/geo/query/acc.cgi?acc=GSE290226]. The mass spectrometry proteomics data have been deposited to the ProteomeXchange Consortium via the PRIDE partner repository with the dataset identifier PXD061385 [http://proteomecentral.proteomexchange.org/cgi/GetDataset?ID=PXD061385]. GC/MS data of targeted metabolites are uploaded to MetaboLights under project accession MTBLS14075 [https://www.ebi.ac.uk/metabolights/MTBLS14075].

The assembled genomes and annotations as well as (intermediate) pipeline results and multiomics data used for analysis in this article, including raw data of figures and additional work to support the findings, can be found on figshare [https://doi.org/10.6084/m9.figshare.26212895]. An interactive genome browser providing access to the predominant chromosome assembly of C. major including gene, pseudogene, and repeat annotations as well as HiC proximity signals and alignments to CANU and the draft assemblies of C. minor s.l. and C. rosea [https://apps.pph.univie.ac.at/jbrowse/]. Source Data are provided with this paper.

Research involving human participants, their data, or biological material

Policy information about studies with human participants or human data. See also policy information about sex, gender (identity/presentation), and sexual orientation and race, ethnicity and racism.

Reporting on sex and gender	*Use the terms sex (biological attribute) and gender (shaped by social and cultural circumstances) carefully in order to avoid confusing both terms. Indicate if findings apply to only one sex or gender; describe whether sex and gender were considered in study design; whether sex and/or gender was determined based on self-reporting or assigned and methods used. Provide in the source data disaggregated sex and gender data, where this information has been collected, and if consent has been obtained for sharing of individual-level data; provide overall numbers in this Reporting Summary. Please state if this information has not been collected. Report sex- and gender-based analyses where performed, justify reasons for lack of sex- and gender-based analysis.*
Reporting on race, ethnicity, or other socially relevant groupings	*Please specify the socially constructed or socially relevant categorization variable(s) used in your manuscript and explain why they were used. Please note that such variables should not be used as proxies for other socially constructed/relevant variables (for example, race or ethnicity should not be used as a proxy for socioeconomic status). Provide clear definitions of the relevant terms used, how they were provided (by the participants/respondents, the researchers, or third parties), and the method(s) used to classify people into the different categories (e.g. self-report, census or administrative data, social media data, etc.) Please provide details about how you controlled for confounding variables in your analyses.*
Population characteristics	*Describe the covariate-relevant population characteristics of the human research participants (e.g. age, genotypic information, past and current diagnosis and treatment categories). If you filled out the behavioural & social sciences study design questions and have nothing to add here, write "See above."*
Recruitment	*Describe how participants were recruited. Outline any potential self-selection bias or other biases that may be present and how these are likely to impact results.*
Ethics oversight	*Identify the organization(s) that approved the study protocol.*

Note that full information on the approval of the study protocol must also be provided in the manuscript.

Field-specific reporting

Please select the one below that is the best fit for your research. If you are not sure, read the appropriate sections before making your selection.

☐ Life sciences ☐ Behavioural & social sciences ☒ Ecological, evolutionary & environmental sciences

For a reference copy of the document with all sections, see nature.com/documents/nr-reporting-summary-flat.pdf

Ecological, evolutionary & environmental sciences study design

All studies must disclose on these points even when the disclosure is negative.

Study description	Genomes of three tropical trees Clusia major, Clusia minor s.l. and Clusia rosea were sequenced.
Research sample	Leaf samples of Clusia major, Clusia minor and Clusia rosea for genome sequencing were obtained from the collection of the Molecular Systems Biology lab at the Department of Functional and Evolutionary Ecology, University of Vienna, including the living collection originally curated by Ulrich Lüttge. Our laboratory maintains a backup of this Clusia collection, with the primary collection still housed at the Botanical Garden of the Technical University Darmstadt, Germany. Cuttings of the entire collection were transferred to the greenhouse of the Molecular Systems Biology lab, Department of Functional and Evolutionary Ecology, University of Vienna and cultivated and propagated there. Initially the greenhouses were located at UZA1, Althanstrasse 14, 1090 Vienna, and as of 2021, the plants were transferred to the new greenhouse at the UBB, Schlachthausgasse 43, 1030 Vienna. Voucher specimens of the cultivated species were prepared following standard herbarium practice and preserved as dried specimens

	and/or flowers in 70% ethanol, and permanently stored in the herbarium of the Natural History Museum Vienna. Digital images of the voucher specimens and photos of the corresponding living plants are available at the international herbarium database JACQ (https://jacq.org). All relevant specimen vouchers including links to virtual herbaria sources are documented in Supplementary Table 1 . Further stress and multiomics experiments with these three species were conducted in climate chambers and an open greenhouse experiment.
Sampling strategy	Leaf samples were collected from all three species in a glashouse (genome sequencing), climate chambers (phenotyping), and open greenhouse (multiomics). If not otherwise stated in the manuscript n= 3 samples were collected.
Data collection	HK and JH extracted HMW gDNA for genome sequencing. GB, HK and AD performed plant physiological phenotyping. HK, JH, TS, LF, SP, FS, FT, AKB and GB conducted greenhouse experiments. MI, SP and JH extracted RNA from Clusia species, JH and HK performed transcriptome data analyses. PK, SP, LF, MB and CP extracted metabolites and performed data acquisition. CP and JH analyzed metabolomics data. FF, LAS, PC, AG and TS performed proteomic data acquisition. FS performed starch assays. HWS, EMT and TS conducted genome size estimation experiments and karyotyping. ADB revised the taxonomy and documented the plant species in the herbarium.
Timing and spatial scale	Plant material for experimentation was generated by producing a total of 48 cuttings from mother plants. Clusia major, C. minor, and C. rosea were propagated in a clonal fashion from those plants, which we used for DNA extractions and genome sequencing. Cuttings were cultivated in the greenhouse for eight months until all plants reached at least a four-leaf stage, and they were then randomly divided into two groups. The experiment was conducted during a sunny summer week in July 2021. For one week, one group was exposed to direct sunlight. There was no precipitation, and the plants were not irrigated. The other group was placed beneath a shaded cultivation table and each pot received 50 mL of water each day, corresponding to 60% field capacity (approx. 30% soil water). Via soil moisture, photosynthetic active radiation (PAR), and temperature loggers (placed in the shade), connected to Arduinos and a Raspberry Pi, we continuously monitored environmental parameters (Supplementary Code, Supplementary Data 9Materials). We selected six uniform cuttings from each plant species based on DLI (three replicates per group). On July 15th 2021, we started sampling leaf tissue on four time points across a 24-hour period, at 4 am, 8 am, 1 pm, and 7 pm, respectively. We used sterile punch pliers with a 5 mm diameter to punch four holes into one leaf of each plant. At each time-point a new leaf section (with respect to the main nerve) was used to reduce the amount of injury related signals. The leaf tissue was transferred into 2 mL centrifuge tubes and was immediately snap-frozen in liquid nitrogen and stored at -80 °C for subsequent multiomics analyzes.
Data exclusions	No data were excluded
Reproducibility	Biological replicates of control and stress treated samples were analysed in all cases showing statistical significant separation where indicated.
Randomization	All measurements are randomized.
Blinding	No blinding

Did the study involve field work? ☐ Yes ☒ No

Reporting for specific materials, systems and methods

We require information from authors about some types of materials, experimental systems and methods used in many studies. Here, indicate whether each material, system or method listed is relevant to your study. If you are not sure if a list item applies to your research, read the appropriate section before selecting a response.

Materials & experimental systems

n/a	Involved in the study
☒	☐ Antibodies
☒	☐ Eukaryotic cell lines
☒	☐ Palaeontology and archaeology
☒	☐ Animals and other organisms
☒	☐ Clinical data
☒	☐ Dual use research of concern
☐	☒ Plants

Methods

n/a	Involved in the study
☒	☐ ChIP-seq
☒	☐ Flow cytometry
☒	☐ MRI-based neuroimaging

Plants

Seed stocks	Clusia trees are part of a collection in the greenhouse of the University of Vienna.
Novel plant genotypes	No novel genotypes
Authentication	No genetic manipulation
